# Synthesis, optimization, and cell response investigations of natural-based, thermoresponsive, injectable hydrogel: An attitude for 3D hepatocyte encapsulation and cell therapy

**DOI:** 10.3389/fbioe.2022.1075166

**Published:** 2023-01-06

**Authors:** Mahnaz Gholami, Maryam Tajabadi, Alireza Khavandi, Negar Azarpira

**Affiliations:** ^1^ School of Metallurgy and Materials Engineering, Iran University of Science and Technology (IUST), Tehran, Iran; ^2^ Transplant Research Center, Shiraz University of Medical Science, Shiraz, Iran

**Keywords:** injectable hydrogel, chitosan, silk fibroin, genipin, glycerophosphate, sodium hydrogen carbonate, thermoresponsive

## Abstract

For the purpose of developing a 3D vehicle for the delivery of hepatocytes in cell therapy, the improved system of crosslinker and new gelling agent combinations consisting of glycerophosphate and sodium hydrogen carbonate have been employed to produce injectable, thermoresponsive hydrogels based on chitosan and silk fibroin. Adjusting the polymer-to-gelling agent ratio and utilizing a chemical crosslinker developed hydrogel scaffolds with optimal gelling time and pH. Applying sodium hydrogen carbonate neutralizes chitosan while keeping its thermoresponsive characteristics and decreases glycerophosphate from 60% to 30%. Genipin boosts the mechanical properties of hydrogel without affecting the gel time. Due to their stable microstructure and lower amine availability, genipin-containing materials have a low swelling ratio, around six compared to eight for those without genipin. Hydrogels that are crosslinked degrade about half as fast as those that are not. The slowerr degradation of Silk fibroin compared to chitosan makes it an efficient degradation inhibitor in silk-containing formulations. All of the optimized samples showed less than 5% hemolytic activity, indicating that they lacked hemolytic characteristics. The acceptable cell viability in crosslinked hydrogels ranges from 72% to 91% due to the decreasing total salt concentration, which protects cells from hyperosmolality. The pH of hydrogels and their interstitial pores kept most encapsulated cells alive and functioning for 24 h. Urea levels are higher in the encapsulation condition compared to HepG2 cultivated alone, and this may be due to cell-matrix interactions that boost liver-specific activity. Urea synthesis in genipin crosslinked hydrogels increased dramatically from day 1 (about 4 mg dl^−1^) to day 3 (approximately 6 mg dl^−1^), suggesting the enormous potential of these hydrogels for cell milieu preparation. All mentioned findings represent that the optimized system may be a promising candidate for liver regeneration.

## Graphical Abstract

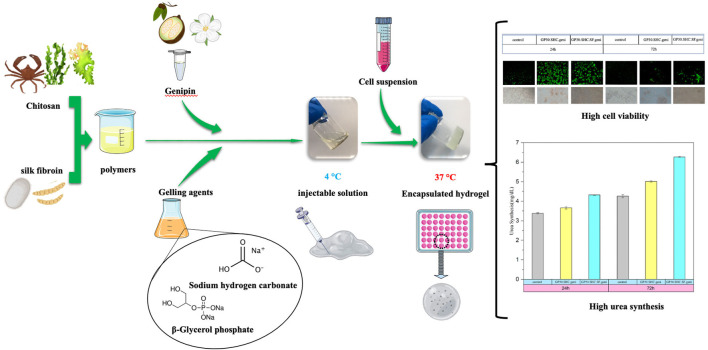

## 1 Introduction

As people age, their organs degenerate due to the normal human growth process. Various disorders and situations can also induce organ failure. Numerous treatments have been proposed, but none have been effective. Some illnesses are not drug-treatable, and in end-stage organ failure, the necessity for immunosuppression in organ transplantation may lead to infections and malignancies. Cell therapy, tissue engineering, and genetic manipulation have been studied to address aging, disease, and tissue and organ shortages (Ashammakhi et al., 2019). Improving cell survival and retention, tissue incorporation, and patient safety during cell therapy administration is essential. The most common cell transplantation methods are intravenous/intraarterial and intra-tissue injections. Most of these methods do not work because the cells that are transplanted die or migrate to the wrong place (De Pieri et al., 2021). When cells are transplanted *via* these routes, only around 1%–20% of them remain viable, which dramatically limits their therapeutic potential (Marquardt and Heilshorn, 2016). Tissue engineering was created to deal with these problems by introducing cells to biomaterials in a more integrated fashion. In particular, natural and synthetic compounds have been utilized to promote stem cell differentiation into target cells, alleviate direct cell delivery problems, and create applicable 3D tissue constructs (Ardeshirylajimi et al., 2018; De Pieri et al., 2021). Interdisciplinary research in tissue engineering attempts to develop biomaterials replacing damaged organs and encouraging cell proliferation (Hashemi et al., 2020).

In the case of liver and other soft tissues, the extracellular matrix (ECM)-derived hydrogel that forms the microenvironment of cell delivers various biochemical signals (Ijima et al., 2019; Lee et al., 2020). Every alteration in the ECM of liver affects its structure and function, highlighting the critical role of the surrounding ECM in preserving ability of hepatocytes to carry out their specialized hepatic roles (Saheli et al., 2018; Ye et al., 2019). When designing hydrogels, it is important to take into account the specific needs of hepatocytes for biocompatibility, biodegradability, thermoresponsiveness, and swelling (Ye et al., 2019). In order to create a microenvironment that is more similar to the liver, 3D scaffolds made from natural biomaterials are preferred (Lee et al., 2020). Anchorage-dependent hepatocytes modulate cell-matrix interactions. The matrix for cultivating hepatocytes should be extremely porous and mechanically stable, allowing nutrition, metabolite, and growth factor diffusion to stimulate vascularization and maintain liver functions (Janani et al., 2018). Encapsulated hepatocytes offer hepatic activities without immunosuppression, since they are shielded from activated immune cells (Iansante et al., 2018; Zhang et al., 2021).

Hydrogels, which emerged in the literature in 1894, are commonly employed in tissue engineering and regenerative medicine as a promising and encouraging candidate biomaterial. Hydrogels are a network of naturally occurring or synthesized hydrophilic polymer chains that are as flexible as natural tissues (Ye et al., 2019). By demonstrating structural similarities to biomacromolecules found in ECMs, hydrogels have played an important role in improving the permeability of oxygen, vitamins, and other water-soluble metabolites and stimulating biological activities (Afewerki et al., 2019). Hydrogels, with their adjustable porosity, changeable transpermeability, and appropriate mechanical properties, not only allow for the delivery of oxygen and nutrients and the release of depleted metabolites but also protect encapsulated cells from cytotoxic molecules, mechanical loads, and immune attacks (Wang et al., 2018). The recently coined phrase “injectable hydrogel” has gradually attracted researchers’ interest in hydrogel-based biomaterials due to its capacity to fulfill biological tasks (Liao et al., 2020). Because of their minimally invasive nature and ability to develop a specific shape, injectable hydrogels might be the next hot topic for surgeons. Injectable hydrogels may also create minor frictional irritations in natural tissue and eliminate the need for complex surgical procedures (Alinejad et al., 2018). To be used in medicine, an injectable hydrogel must be robust enough to withstand the stresses of its intended application but flexible enough to reach the correct site quickly (Pettinelli et al., 2020). Polysaccharides, proteins, and peptides, i.e., natural polymers, are often employed to create injectable hydrogels. Among them, as a result of their superior water solubility, biocompatibility, and biodegradability, polysaccharides are commonly employed to create hydrogels for injection (Thambi et al., 2016; Rijal et al., 2017).

As a deacetylated derivative of chitin, chitosan (Cs) is composed of β-(1-4) linked D-glucosamine and N-acetylglucosamine groups, the distribution of which varies with the degree of deacetylation (Jiang et al., 2015; Ahmed et al., 2018; Islam et al., 2020). Since chitosan mimics glycosaminoglycan, the foundational element of the ECM, it has been used as a scaffold for tissue engineering in recent years (Ardeshirylajimi et al., 2018). Temperature-responsive systems based on chitosan, a class of hydrogel systems that change their liquid state in response to the external temperature, are being investigated to help cell proliferation in tissue regeneration (Bhattarai et al., 2010; Saravanan et al., 2019). Chenite et al. (2000) and Pankongadisak and Suwantong (2019) previously introduced injectable thermogelling Cs/β-glycerol phosphate disodium salt (Cs/GP) hydrogels, which have a fluid shape at low temperatures but gel at 37°C. Glycerophosphate (GP) is generally identified in the body and utilized as a phosphate supply when phosphate absorption is unbalanced. Theoretically, the phosphates in GP salt neutralize the ammonium groups in chitosan, enabling greater hydrophobicity and hydrogen bonding between chitosan chains at higher temperatures. While the combination is still liquid at room temperature, it begins to gel around 37°C (Thambi et al., 2016).

As mentioned above, the ideal injectable hydrogel for tissue regeneration has a short gelation period, controlled degradation, and desirable pH stability. The concentration of GP may be adjusted to influence degradation and gelation durations, the greater the concentrations, the faster gelation. However, it has been shown that higher GP degrees cause hypertonicity and cell death. In order to decrease the concentration of GP, sodium hydrogen carbonate, NaHCO_3_, is used, which results in improved thermal stability, reduced cytotoxicity, and shorter gelation times (Assaad et al., 2015; Saravanan et al., 2019).

Biocompatibility, hydrophilicity, and acceptable mechanical properties may all be achieved by combining silk fibroin (SF) and Cs (Li et al., 2018). Silkworm silk has two major components, i.e., silk fibroin and silk sericin; among them, silk fibroin has long been used in tissue engineering and regenerative medicine as a natural polymer, resulting in enhanced degrees of interconnection and increased surface area for cell attachment (Floren et al., 2016; Sun et al., 2016; Panjapheree et al., 2018). Fibroin is made up of a repeating amino acid sequence that includes glycine, serine, and alanine and could be obtained by removing cytotoxic silk sericin (Celikkin et al., 2017).

The crosslinkers, such as glutaraldehyde or genipin, have modified polymer components to stabilize the therapeutic system in a biological environment. Primary amines in protein-rich domains, such as chitosan and silk, may react with these crosslinkers (Sun et al., 2016). From the fruits of Gardenia jasminoides J. Ellis, genipin, a colorless monoterpene of the iridoid class, could be extracted. Genipin, rather than glutaraldehyde, is preferred to crosslink the hydrogel system because of its low toxicity, which allows it to be used directly in living tissue (Mirzaei et al., 2014; Neri-Numa et al., 2017). Thus, crosslinking agent based on genipin increases the mechanical characteristics of the scaffold while also helping to promote cell survival (Kwon et al., 2015).

The present study aims to investigate new formulations that enable the remarkable increase of mechanical properties and cytocompatibility of chitosan thermogels and rapid gelation. This was achieved by incorporating silk fibroin into the polymer section of hydrogel and combining GP with another weak base, i.e., sodium hydrogen carbonate (SHC), to reduce the concentration of the final gelling agent compound. Moreover, the hydrogel system, composed of chitosan-silk fibroin and gelling agent compounds, was crosslinked with genipin. Through optimizing parameters like pH, mechanical properties, gelation time, swelling behavior, biodegradability, and hemocompatibility, this research seeks to identify the best hydrogel to use as an injectable scaffold for minimally invasive therapeutic tissue engineering applications.

## 2 Experimental

### 2.1 Materials and methods

Chitosan (Cs, CAS NO. 9012-76-4), β-Glycerol phosphate disodium salt pentahydrate (C_3_H_7_Na_2_O_6_P·_5_H_2_O, GP, CAS No. 13408-09-8), Sodium hydrogen carbonate (NaHCO_3_, SHC, Cas No. 144-55-8), and also reagents that include acetic acid(AA), lithium bromide (LiBr), sodium carbonate (Na_2_CO_3_) and dimethyl sulfoxide (DMSO) were obtained from Sigma-Aldrich. Genipin (C_11_H_14_O_5_, Geni, CAS No. 6902-77-8) was provided by Challenge Bioproducts Co. Ltd. (Touliu, Taiwan).

The cocoons of the silkworm (Bombyx mori) were provided by the University of Gilan. Dialysis bags (MWCO 12000) were supplied by Sigma-Aldrich. Dulbecco’s Modified Eagle’s Medium (DMEM), Dulbecco’s Modified Eagle Medium/Nutrient Mixture F-12 (DMEM/F-12), penicillin-streptomycin antibiotics, trypsin-ethylenediaminetetraacetic acid (Trypsin- EDTA) and Fetal bovine serum (FBS) were purchased from Gibco™. 3-(4,5-Dimethylthiazol-2-yl)-2,5-diphenyl- tetrazolium bromide (MTT) were purchased from Sigma-Aldrich Co.

### 2.2 Hydrogel fabrication

#### 2.2.1 Polymer solutions

##### 2.2.1.1 Silk fibroin

The following instructions describe the preparation of silk fibroin solution that includes a degumming process. The degumming of fibers is performed to remove the glue-like sericin proteins of Bombyx mori silkworm cocoon fibers (Cárdena-Pérez et al., 2017).

The cocoons of Bombyx mori were boiled in a 0.05% Na_2_CO_3_ aqueous solution for 30 min at 98°C–100°C in triplicate in accordance with the technique mentioned somewhere else (Yin et al., 2017; Tao et al., 2021). The boiled cocoons were rinsed with deionized water throughout the process to eliminate the glue-like sericin proteins. The cleansed cocoons were then left to dry for almost half a day at 60°C. Degumming and drying the silk fiber allowed for a smoother dissolution in a 9.3 M LiBr solution at 60°C for 1 h, yielding the purified aqueous silk fibroin solution. As a subsequent step, the SF solution with a 10% concentration (w/v) was dialyzed towards distilled water for 3 days at room temperature using a dialysis membrane (M_W_ cutoff: 12000). The last contaminants of silk fibroin solution were removed by centrifuging at 4,500 rpm for 10 min at 4°C. To be employed in subsequent formulations, silk fibroin has been lyophilized and then dissolved in deionized water to generate a 1% (w/v) SF solution (Li et al., 2018).

##### 2.2.1.2 Chitosan

The chitosan powder (Cs) was dissolved in distilled water with 1% (by volume) acetic acid to produce a solution with a final chitosan concentration of 1.3% (w/v). The resulting solution was stirred at room temperature overnight. While stirring, 1 ml of SF 1% was then introduced to the chitosan solution so that the mixture of Cs/SF with a weight ratio of 1:1 was produced. The temperature of this solution was reduced to 4°C.

#### 2.2.2 Gelling agent solutions

In terms of gelling agents (GA), GP alone, GP-SHC, or GP-SHC-Geni was used (genipin as crosslinker). The three types of gelling agent solutions are prepared by first preparing 30% (w/v) GP solution in distilled water, stirring in ice, then adding SHC powder with additional stirring until a final concentration of 0.05 M SHC is reached. The genipin solution was made by dissolving 10 mg of genipin in 1 ml of distilled water. This solution was then added to the GA solution until the final genipin concentration was 50 μg/ml. Gelling agents were filtered *via* 0.2 μm filters, sterilized, and then kept at 4°C.

#### 2.2.3 Preparation of hydrogels for physicochemical characterization

To prepare each hydrogel at 4°C, dropwise additions of GA solutions were made to each of the sterilized polymer solutions with a volume-to-volume ratio of 1:1 (v/v). Further stirring was done to achieve homogeneous solutions. A list of all synthesized samples can be found in Table 1. Table 2 shows the samples chosen for further research, representing the optimal pH and gelling time. Hydrogels are named based on their composition. Cs concentrations in all formulations were maintained at 1.3% (w/v); therefore, the name of the resulting hydrogels will be determined using the final concentration of the GA components. As an example, GP30.SHC.SF corresponds to a gel composed of 1.3% (w/v) of Cs, 0.05 M SHC, 1% (w/v) of SF and 30% GP Table 2.

**TABLE 1:** The preliminary studys’ hydrogel compositions.

Sample number	Cs(%)	GP(%)	SF(%)	SHC(M)	Geni(µgr/ml)	Gel formation	Description
1	1.3	0	0	0.075	0	+	Basic pH
2	1.3	0	0	0.05	0	—	—
3	1.3	10	0	0	0	—	—
4	1.3	30	0	0	0	+	Lengthy gelation time
5	1.3	50	0	0	0	+	Appropriate gel
6	1.3	50	0	0.05	0	+	Low fluidity in the solution phase
7	1.3	50	0	0.075	0	+	Early gel formation
8	1.3	30	0	0.05	0	+	Appropriate gel
9	1.3	30	0	0.075	0	+	Early gel formation
10	1.3	10	0	0.05	0	—	—
11	1.3	10	0	0.075	0	+	Basic pH
12	1.3	30	1	0.05	0	+	Appropriate gel
13	1.3	30	2	0.05	0	+	Low fluidity in the solution phase
14	1.3	30	1	0.05	25	+	No color shift
15	1.3	30	1	0.05	50	+	Appropriate gel
16	1.3	30	0	0.05	25	+	No color shift
17	1.3	30	0	0.05	50	+	Appropriate gel

**TABLE 2:** Composition, pH, and gelling time of optimal synthesized hydrogels. The hydrogels had a Cs concentration of 1.3% (w/v).

Sample	Cs(%)	GP(%)	SHC)M(	SF(%)	geni(µgr/ml)	PH	Gelation time(Sec)
GP50	1.3	50	0	0	0	6.78 ± .02	382.3 ± 8.7
GP30.SHC	1.3	30	0.05	0	0	7.11 ± .09	230.6 ± 2.5
GP30.SHC.SF	1.3	30	0.05	1	0	7.31 ± .03	199.3 ± 4.1
GP30.SHC.SF.geni	1.3	30	0.05	1	50	7.34 ± .01	230.0 ± 3.2
GP30.SHC.geni	1.3	30	0.05	0	50	7.20 ± .02	220.0 ± 1.6

#### 2.2.4 Preparation of hydrogels for cell encapsulation

Two-step procedure was used for the cell encapsulation using two optimized hydrogel formulations, namely GP30.SHC.SF.geni and GP30.SHC.geni. First, dropwise additions of 2X concentrated filter-sterilized (0.22 µ) gelling agent to the stirred polymer solution on the ice were performed. Under aseptic conditions, the resultant mixture was stirred for 15 min. The cell suspensions in culture media, with concentrations of 5×10^5^ cells/ml, were then inoculated into the polymer solutions at a volume ratio of 3:1 (3 for the cell suspensions and one for the polymer-GA). In each well of a 48-well plate, a volume of 500 μl gel solution containing cells was left to gel for 30 min at 37°C. After that, 400 μl of culture medium was added to the cell/hydrogel specimens, and they were incubated for another 24 h. The control was a culture medium with cells at the bottom of the 48-well plates (no scaffold).

### 2.3 Physicochemical characterization

#### 2.3.1 Gel time and pH determination

For the aim of evaluating gel formation, an inverted tube test was performed. 1 ml of prepared polymer solutions mixed with gelling agents was kept at 4°C in 5 ml vials and kept in a 37°C water bath after 5 min of mixing in an ice/water bath. The time required for the sol-gel transition was measured by turning the vial upside down. The gelation time refers to the time when the gel did not flow. After incubating the hydrogels for 24 h at 37°C, they were pressed through 0.45 µ filters to yield an entrapped solution of hydrogel filtrates (Alinejad et al., 2018). Their pH was measured using a Denver Instrument UltraBasic pH meter.

#### 2.3.2 Fourier transform infrared spectroscopy

In order to explore the effect of different gelling agents and crosslinker on the synthesis of hydrogels, the Fourier transform infrared (FTIR) spectra of freeze-dried samples of hydrogels were used. FTIR spectra were obtained using a Bruker TensorII FTIR spectrometer at wavenumbers ranging from 400 to 4,000 cm^−1^.

#### 2.3.3 SEM

A scanning electron microscope (SEM) was used to analyze the morphology and microstructure of the chitosan hydrogels. For this purpose, after 24 h of gelation, samples were frozen overnight at –20°C and then lyophilized for 24 h under the vacuum condition. Following dehydration, the samples were divided with a scalpel blade, gold-sputter coated, and examined using a scanning electron microscope (SEM; Hitachi S-3600).

#### 2.3.4 Mechanical properties

After either 1 or 24 h of gelation at 37°C, 800 μl of each sample was deposited onto a 24-well plate and put in an incubator to assess the hydrogels’ mechanical characteristics. Gel samples were prepared by using a sample punch to create cylinders with a diameter of 10 mm and a height of around 8 mm. A Bose ElectroForce^®^ 3,200 instrument (Bose Corporation, United States) with a 22 N load cell is used to apply progressive compression up to 50% axial deformation (5%–50%) at a rate of 100% deformation/min, allowing for a complete characterization of the hydrogels. The Young secant moduli, defined as the slope of a line connecting the point of zero strain to a point at a specific deformation, were determined using displacement and load measurements.

#### 2.3.5 Swelling measurement

Lyophilized samples were used to assess the swelling properties of the hydrogels. Each gel was immersed in PBS (phosphate buffer solution) at room temperature for a predetermined period of time to achieve its equilibrium swelling condition. After that, the hydrogels were delicately blotted using filter paper to wipe away any remaining PBS. Afterward, the gels were weighed (W_wet_). The samples were re-immersed in fresh PBS. The swelling percentage was calculated using Eq. 1. All experiments were carried out three times (Alinejad et al., 2018).

$$Swelling\;ratio=\frac{\left(W_{wet}-W_{dry}\right)}{W_{dry}}\times 100$$ (1)

W_dry_ is the initial mass of the lyophilized sample.

#### 2.3.6 *In vitro* biodegradation study

For 4 weeks, at 37°C, in a pH 7.4 buffer solution, hydrogels were submerged in a 1000 U/ml lysozyme solution. Lysozyme concentrations like this, seen in human serum, may physiologically mimic the *in vivo* degradation process (Brouwer et al., 1984). The solution was refreshed every day. Hydrogels were taken out of the medium at regular intervals, gently dried using filter paper to remove surface water, and then weighed. Through the use of Eq. 2, the degree of *in vitro* degradation was quantified as a percentage of weight loss (Song et al., 2018).

$$Weight\;loss\;\left(\%\right)=\frac{\left(W_{i}-W_{t}\right)}{W_{i}}\times 100$$ (2)

Here, W_i_ and W_t_, respectively, denote the initial weight and the final weight of the samples.

### 2.4 Biological evaluation

#### 2.4.1 MTT assay

The MTT assay was used to assess the *in vitro* cytotoxicity of the scaffolds in a human fibroblast cell line. An extraction test based on the ISO 10993-5 Standard (Kane et al., 2009) was used to assess the cytotoxicity of gel. Because fibroblast cells are the major cellular components of connective tissues, they are commonly utilized in biomaterial cytotoxicity investigations (De Souza et al., 2009). Fibroblast cells were cultured in DMEM/F-12 medium supplemented with 10% (v/v) heat-inactivated fetal bovine serum (FBS) and 1% (v/v) penicillin-streptomycin (100 U/ml penicillin G and 100 mg/mL streptomycin) at 37°C, under 5% CO_2_.

This fibroblast cell line was expected to grow in a monolayer in a tissue culture flask that was kept at 90% relative humidity and incubated at 37°C. The media was changed every 3 days, and once the cells attained confluence, they were detached using a 0.25% trypsin/EDTA solution. Cells were counted using a hemocytometer and seeded at a density of 7,000 cells/well in 96-well plates for the 24, 48, and 72-h MTT tests.

The gels were immersed in a culture medium at 1 ml per 1 g of sterilized hydrogels weight extraction ratio and kept at 37°C in a humidified environment of 5% CO_2_ for 48 h. After 24 h, growth media was aspirated and replaced with 100 μl of 25, 50, and 100% concentrations of extraction medium produced based on the extract dilution method of cytotoxicity. Cell viability was assessed using the MTT test method after 24, 48, and 72 h of incubation.

Each well was filled with 100 μl of 5 mg/ml thiazolyl blue tetrazolium bromide solution (in culture media), and the plates were incubated for 3–4 h. MTT solution was taken away after incubation and substituted with 100 μl of DMSO. Finally, cell viability was determined using a Spectra Max Plus microplate reader and optical absorbance at 570 nm (Molecular Devices, CA, United States). Cells cultured with just a medium were used as a control, which was considered 100% cell viability.

#### 2.4.2 Hemolysis

The degree to which the hydrogels caused hemolysis was determined by measuring the amount of hemoglobin that was released into the solution phase from hydrogel-exposed erythrocytes in whole blood. The process for making thermosensitive hydrogels was the same as that utilized for microscopic examination.

0.2 ml of anticoagulated whole blood was added to 10 ml of:

1) 0.9% NaCl solution comprising various hydrogel samples (0.25 g), allowing the samples to be soaked in an anticoagulant blood and saline solution for 120 min at 37°C with gentle agitation.
2) Physiological saline solution and distilled water served as negative and positive controls, respectively.

The samples were incubated for 2 h and centrifuged at 1,000 rpm for 10 min, and the absorbance of the supernatants from each tube was measured at 545 nm in a spectrophotometric plate reader. The tests were performed in triplicate on the samples. Eq. 3 was utilized to calculate the hemolysis ratio.

$$Rate\;of\;hemolysis\left(\%\right)=\frac{{OD}_{sample}-{OD}_{negative}\;}{{OD}_{positive}-{OD}_{negative}}\times 100$$ (3)

#### 2.4.3 Cell viability assessment in hydrogels

HepG2 cells (human hepatocyte-like cell line) were cultured in Dulbecco’s modified Eagle’s medium (DMEM) supplemented with 10% fetal bovine serum, 100 U/ml penicillin, and .1 mg/ml streptomycin at 37°C in a CO_2_ incubator. At 85% confluency, cells were removed from culture dishes using the trypsin-EDTA treatment and then suspended in culture media. Using moderate centrifugation, a cell pellet was formed.

The viability of cells after 24 h of entrapment in the hydrogel was determined using a fluorescence assay that simultaneously identifies live and dead cells using 5 × 10^–2^ μg/ml fluorescein diacetate (green) and propidium iodide (red), both from Sigma-Aldrich, in sterile PBS. The staining solution was applied to the cell-encapsulating hydrogels for 5 min at 37°C, and the cells were observed using an Olympus fluorescent microscope with a digital camera.

#### 2.4.4 Urea synthesis

For the purpose of determining whether or not the encapsulated construct enhances hepatic cell functionality, the urea production level of HepG2 was measured. The encapsulated cells (HepG2 cells in blend gels) were put into a 24-well plate. The medium was changed regularly every day. Following each specific time interval (days 1 and 3), media from 3D culture systems was collected and stored at −80°C before being measured. The urea synthesis rate was measured using the commercial urea UV kit (Pars Azmon, Iran) according to the manufacturer’s protocol. Absorbance at 430 nm was determined after incubating an equivalent volume of working reagents with the medium for 1 h at room temperature in a dark environment (MD SPECTRAMAX 190). A minimum of three replicates were conducted for each sample. The results were presented as mg dL^−1^ and compared to a standard curve.

#### 2.4.5 Statistical analysis

At least three independent replicates of each experiment were conducted. The results of this investigation were presented as the mean SD of the obtained data (SD).

## 3 Result and discussion

### 3.1 Physicochemical characterization

#### 3.1.1 Gel formation of hydrogel

**FIGURE 1:**
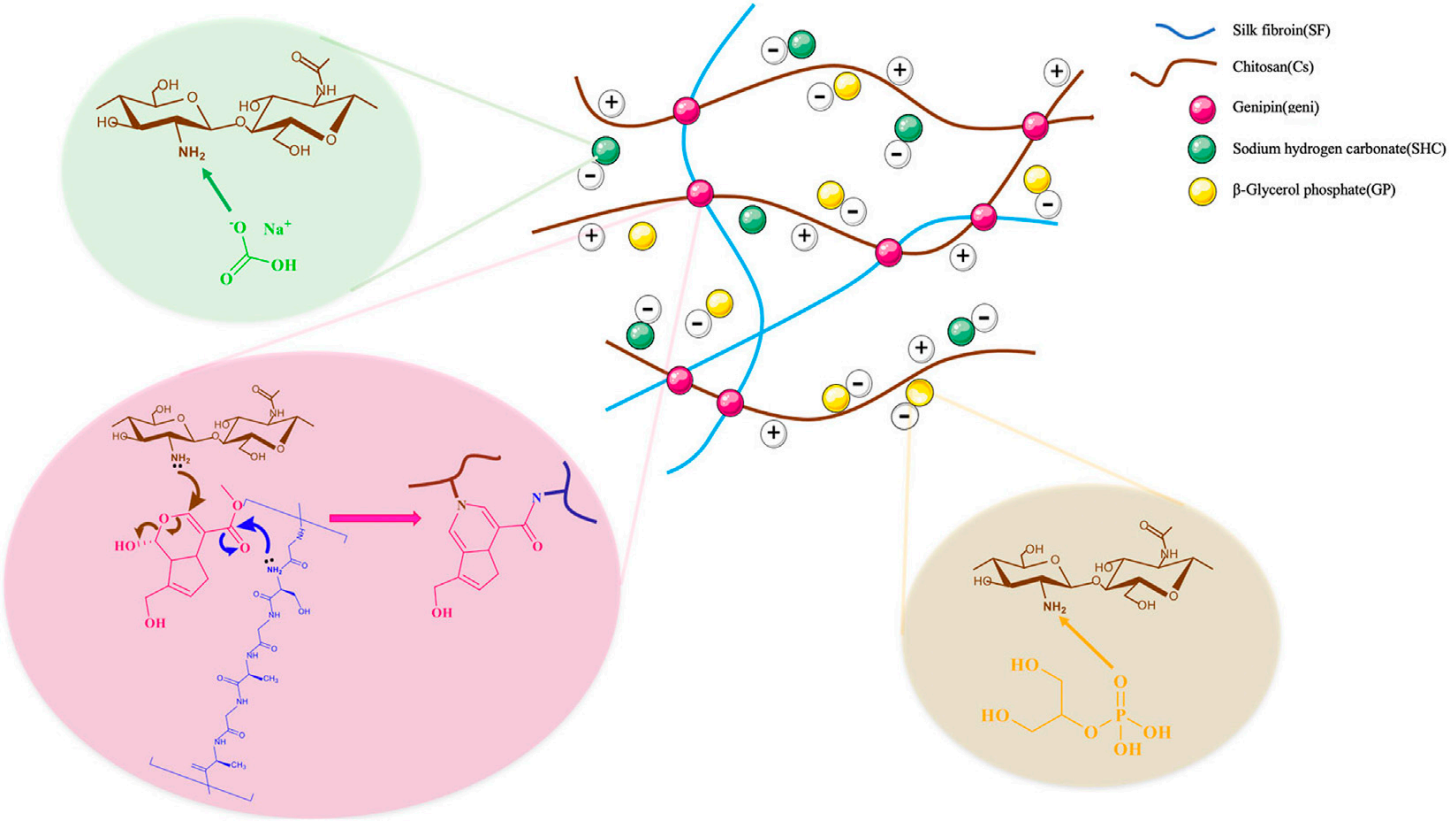
Illustrates the mechanism of production of the hydrogel network composed of polymer, and gelling agents, containing chitosan, silk fibroin, glycerophosphates, sodium hydrogen carbonate, and genipin as crosslinker. The electrostatic interactions between the positive charge of amine group of the polymer and the negative charge of the gelling agent are involved in the gelation mechanism. Between the polymer chains, there are additional hydrophobic interactions and hydrogen bond formation.

The process of gel formation, as seen in Figure 1, will be thoroughly explained below. Since the -NH_2_ groups are protonated in acidic environments, chitosan becomes a polyelectrolyte. Aqueous solution of chitosan solubility is accomplished through the protonation of its amine groups in acidic conditions. The condition of ionization was expressed by the equilibrium reaction, Eq. 4 (Rinaudo et al., 1999):

$${Cs-NH}_{2}{+H}_{3}O^{+}↔{\;Cs-NH}_{3}^{+}{+H}_{2}O$$ (4)

Physical linkages between the macromolecular chains of the polymers are formed when the positive charge density on chitosan chains is reduced, enabling the creation of physical hydrogels. As gelling agents, GP and SHC do not operate as crosslinkers but rather alter the quality of -CH chain interactions and entanglements by influencing the amount and rate of amino group deprotonation upon gelation (Ceccaldi et al., 2017).

At a pH of 6.2 or so, the dissolved chitosan might potentially stay stable in the solution. The production of a hydrated gel-like precipitate is consistently induced when chitosan aqueous solutions are neutralized to a pH greater than 6.2 (Chenite et al., 2000). To be clear, CS is not a thermosensitive polymer on its own; however, Chenite et al. (2000) and Supper et al. (2014) showed that by adding glycerophosphate (GP) to a CS solution, the polymer becomes thermoresponsive at physiological pH. It was mentioned that temperature plays an important role in this particular gel formation (Nilsen-Nygaard et al., 2015).

The influencing factors in the gelling process of the Cs-GP system have been explained as follows as a result of Cho et al. (2005) and Filion et al. (2007) studies. Several interactions may be contained in the gelation of chitosan-GP system, including electrostatic repulsion, ionic crosslinking, hydrophobic effect, and hydrogen bonding interactions. GP, a weak base (pKa 6.65 at 25°C), can raise the pH of chitosan solutions to near-neutral levels. Chitosan would be just soluble in acidic solutions, and when heated up to 37°C, it dissolved in glycerophosphate solutions at a pH close to neutral, resulting in a sol-gel transition. Glycerophosphate salt is an excellent proton receptor, and its temperature-insensitive pKa is close to that of chitosan (Cho et al., 2005). By increasing the temperature of a glycerophosphate and chitosan solution, protons were transferred from the chitosan to the GP, neutralizing the solution and reducing the electrostatic repulsion between the chitosan molecules. This allowed the hydrophobic and hydrogen-bonding interchain forces to initiate physical crosslinking, which resulted in gel formation (Filion et al., 2007). As mentioned above, GP may prevent electrostatic repulsion between chitosan molecules due to its negative charge in the solution. Hydrophobic (–CH_3_) and hydrogen bonding favorable groups (-OH, -NH, and –C=O) are present in chitosan, allowing three-dimensional networks to form (Cho et al., 2005).

To neutralize the chitosan solution and reduce the GP concentration from 60% to 30%, NaHCO_3_ can be used as a buffer. Although NaHCO_3_ did not react with hydrophobic (-CH_3_) or hydrogen bonding-favoring groups (-OH, -NH, and -C=O), it did react with the acid in the chitosan solution and partially replaced GP to neutralize the solution. Thus, the hydrogel system gels faster when NaHCO_3_ is used as a buffer to neutralize the chitosan solution and lower the GP concentration (Huang et al., 2011). It should be noted that employing the weak SHC base as a gelling agent results in CO_2_ production when mixed with the acidic chitosan solution, as described below (Assaad et al., 2015).

$$\left(1\right)\;{Cs-NH}_{2}{+H}^{+}↔{\;Cs-NH}_{3}^{+}$$ (5)

$$\left(2\right)\;H^{+}{+HCO}_{3}^{-}↔H_{2}{CO}_{3}\to H_{2}{O+CO}_{2}$$ (6)

$$\left(3\right)\;{Cs-NH}_{3}^{+}{+HCO}_{3}^{-}↔{Cs-NH}_{3}^{+}{\;/\;HCO}_{3}^{-}\to {CH-NH}_{2}{+H}_{2}{O+CO}_{2}$$ (7)

At 37°C, Eq. 6 proceeds between the H^+^ related to amine group of chitosan and the HCO_3_^−^ of the SHC salt. The reaction speeds up when the concentration of SHC rises, but the pH of the compound falls outside of the physiological range.

The physical properties of the scaffold could be enhanced by the beta-sheet region of SF content (She et al., 2008). Incorporating SF into a hydrogel system may alleviate steric hindrance between CS molecules and promote CS-gelling agent interaction, allowing for a quick gelation rate. Because hydrogen bonds can occur between the amino groups of CS and SF in these hydrogel systems at low temperatures, or water molecules can create hydrogen bonds with SF, the hydrogen bonding interaction was reduced as the temperature was raised. Meanwhile, water molecules have been eliminated from the molecular chains, allowing the hydrophobic CS and SF chains to migrate and entangle with each other (Pankongadisak and Suwantong, 2019).

Biomaterials containing amino groups, such as Cs and SF, have been crosslinked with Genipin. Strong intermolecular interactions and an improvement in the features of the resultant scaffold with regard to cell adhesion were supplied by the introduction of SF to Cs coupled with genipin crosslinking, which is a gentle reaction since they involve molecules containing amino groups (Silva et al., 2008). Crosslinks between primary amine groups were formed as a result of two reactions that transpired at distinct rates. The fastest reaction was a nucleophilic attack on genipin by a primary amine group, which led to the production of a heterocyclic genipin compound linked to the amine group of polymer. The second, slower reaction was the nucleophilic substitution of ester group of genipin to generate a secondary amide link with polymer (Butler et al., 2003).

Finally, by varying the proportion of polymers in the hydrogel composition, as well as the amount of polymer to gelling agents and the use of chemical crosslinkers, hydrogel scaffolds with various properties can be created that can be used in a wide range of tissue engineering applications, including hard and soft tissues, as well as drug delivery.

#### 3.1.2 Gel time and pH determination

As a consequence, from all of the designed samples (formulated in Table 1), hydrogel samples with the proper pH and gelation time, along with their components and concentrations, are identified and listed in Table 2.

The first crucial consideration in employing injectable hydrogels as a drug or cell carrier is to ensure that the pH is close to that of the human body and also that the gelation duration is optimal. The appropriate gel time for the hydrogel should neither be so quick that it gels prior to injection and reaching the target tissue nor should it be so lengthy that the solution does not gel and is translocated to the surrounding tissues after the hydrogel solution reaches the specified tissue. According to the MTT test, which will be further explained, the hydrogel itself is harmless and non-toxic, and it is also necessary to keep the pH of hydrogel within the body’s physiological pH range, so that cells can remain within it.

**FIGURE 2:**
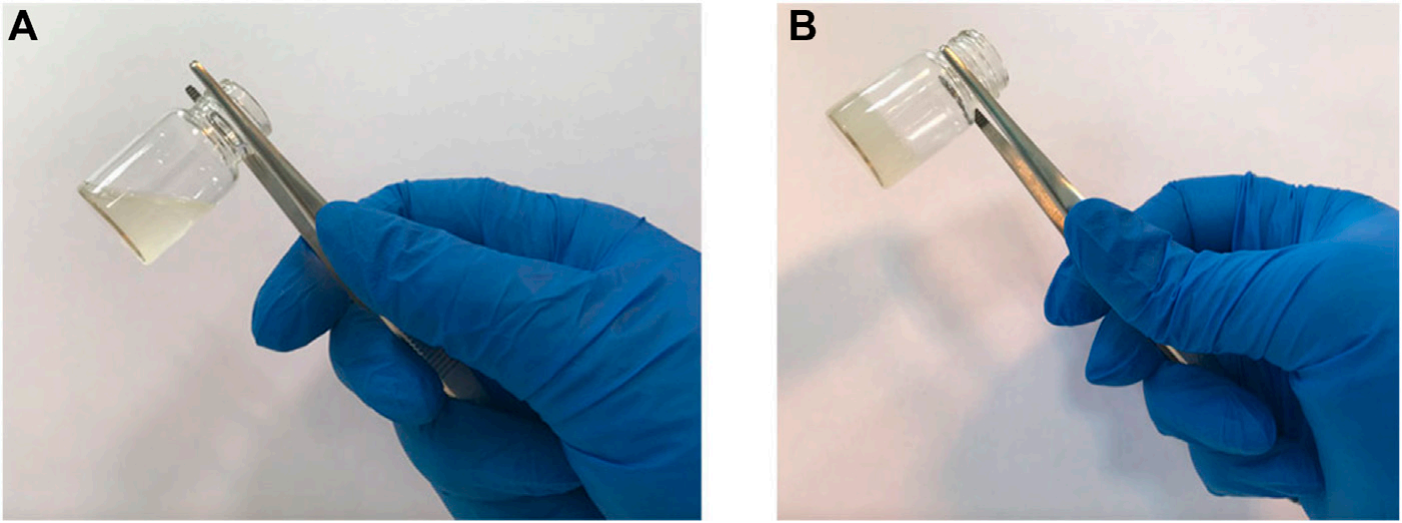
Inversion tube test of the samples to determine the gel time. **(A)** hydrogel at room temperature, and **(B)** hydrogel after incubation at 37°C.

A typical tube inversion test from a hydrogel sample can be seen in Figure 2. After being exposed to 37°C for a certain time period, the sample solution, which flows in a fluid form at room temperature, has the capacity to transfer to the gel form. When SHC is added to chitosan, carbon dioxide is produced, elevating the pH of scaffold over the physiological limit and rendering it unusable in medical settings. However, when utilizing the GP30.SHC sample, which contains both SHC and GP, gel times are shortened, and pH is maintained within the physiological range.

**FIGURE 3:**
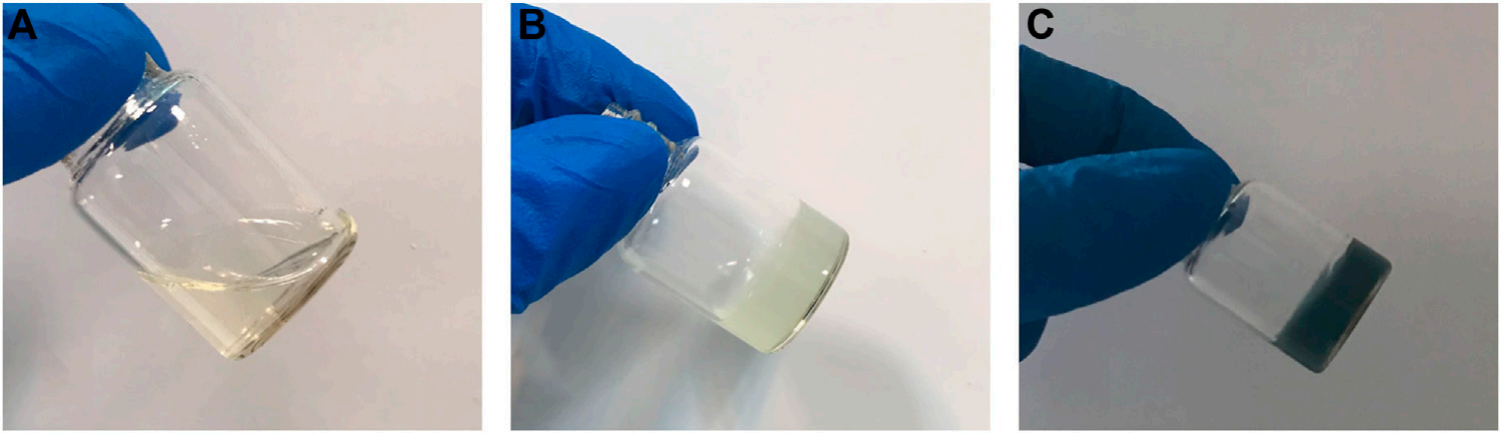
Hydrogel containing genipin in **(A)** room temperature **(B)** after incubation in 37 °C for 5 min and **(C)** after 24 h.

At 37°C, the effect of genipin on the hydrogel could be observed when the hydrogel turns blue, Figure 3. Actually, by inducing crosslinking in the structure of the hydrogel, genipin increases the mechanical properties of scaffold, and its usage has negligible effects on the gel time. Hydrogels containing these substances, as shown in Table 2, gel at a comparable rate and are temperature sensitive, like the body’s physiology, leading to high cell viability in scaffolds.

#### 3.1.3 FTIR

**FIGURE 4:**
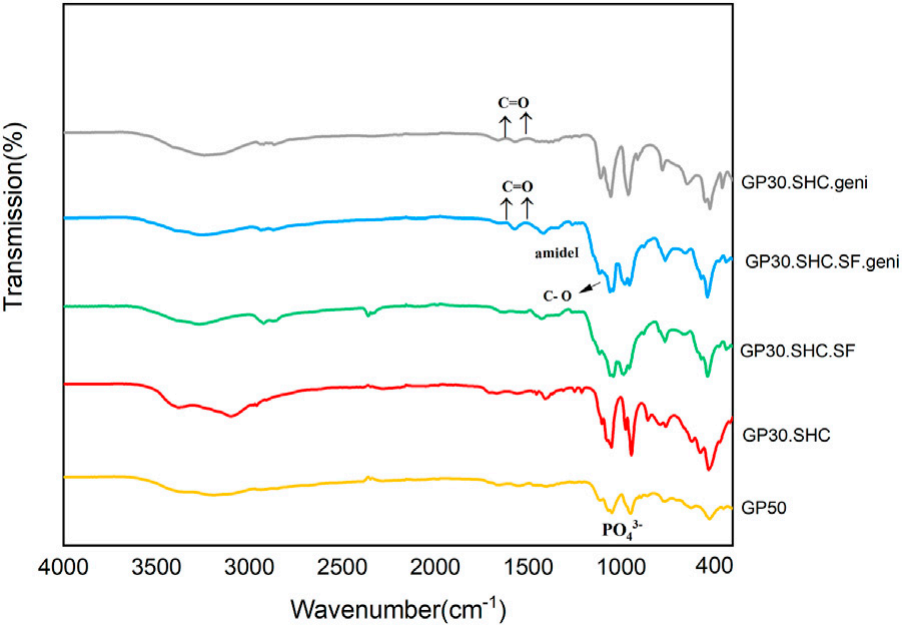
Fourier transform infrared (FTIR) spectra of chitosan hydrogels and the corresponding hydrogels.

It was necessary to conduct FTIR analysis on hydrogel samples in order to determine whether functional groups related to hydrogel-forming compounds were present. The FTIR spectra of hydrogel samples are displayed in Figure 4. The 1,518 cm^−1^ signal in the GP50 spectra was attributed to protonated amino groups due to the presence of acetic acid in the solvent (Song et al., 2018). The absorption band at 1,630 cm^−1^ was assigned to the C=O stretch of the amide bond (Song et al., 2018). The broad band seen between 2,800 and 3,600 cm^−1^ was attributed to GP salt hydroxyl and alkyl group absorption. The strong absorption bands observed at 975 cm^−1^ were associated with the PO_4_^3-^ (Pankongadisak and Suwantong, 2019).

It is possible that the decomposition of SHC in the acidic Cs solution reduces the intensity of the SHC bands at 1700–1,600 cm^−1^ and 1,400–1,300 cm^−1^ (Assaad et al., 2015; Saravanan et al., 2019). Intermolecular interactions between SF and CS during the blending process resulted in absorption bands at 1,627 cm^−1^ (amide I) and 1,521 cm^−1^ (amide II), as well as absorption bands at 1,060 cm^−1^ due to C–O stretching. It is worth noting that the combined ratio of the fibroin silk and chitosan linkages influences their specific adsorption (Li et al., 2018).

As a result of the stretching vibrations of C = O, genipin exhibits characteristic absorption bands at 1,645 and 1,679 cm^−1^ (Song et al., 2018). After crosslinking reaction of genipin in two hydrogel samples GP30.SHC.SF.geni and GP30.SHC.geni, the C = O peaks at 1,615 and 1,531 cm^−1^ show a shift related to the formation of amide bonds. It can be stated that the formation of amide bonds here is the result of a reaction between the amine groups of Cs-SF and the ester group genipin (Song et al., 2018).

#### 3.1.4 SEM

**FIGURE 5:**
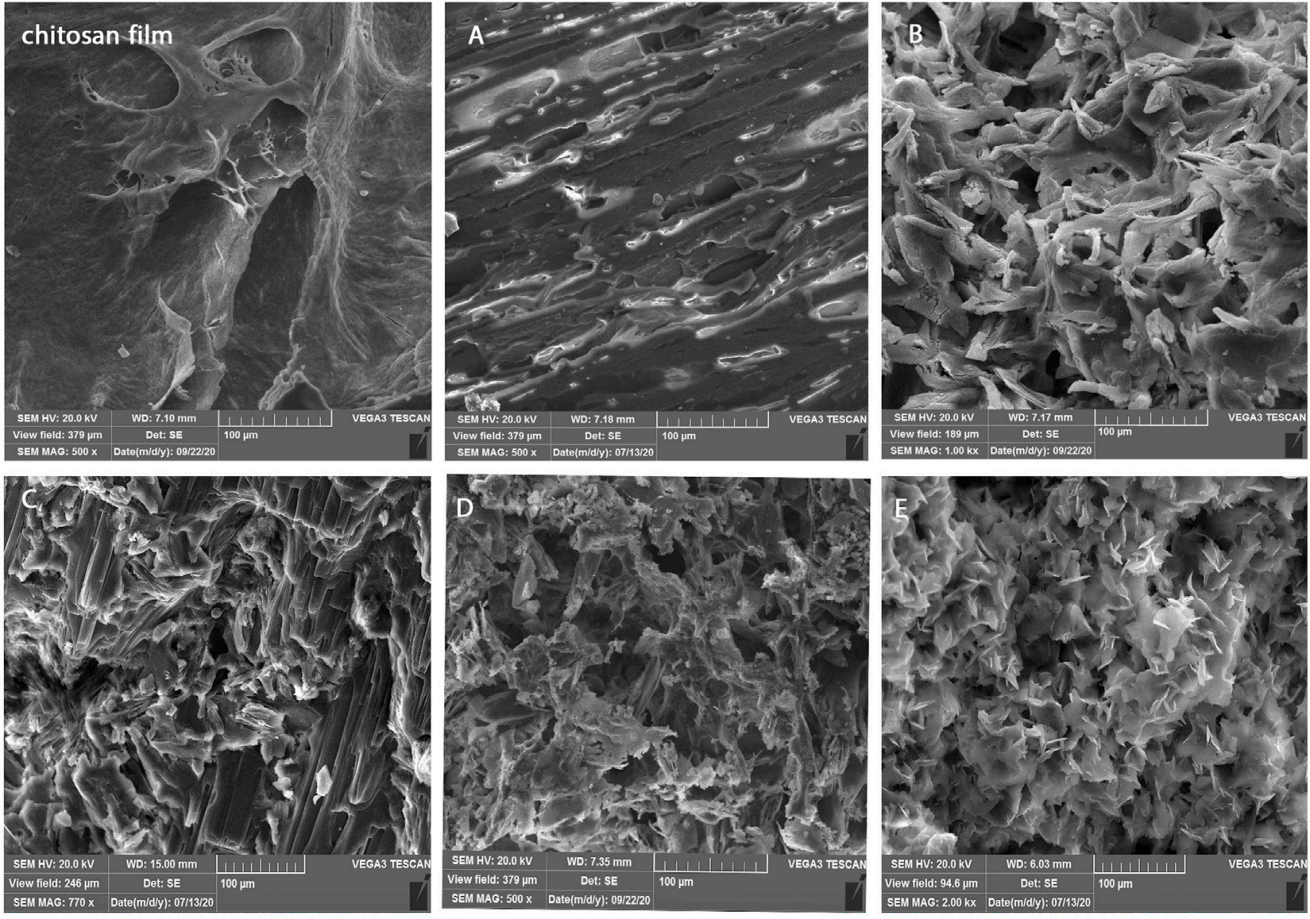
Morphology of chitosan films and hydrogels included **(A)** GP50, **(B)** GP30.SHC, **(C)** GP30.SHC.SF, **(D)** GP30.SHC.SF.geni, and **(E)** GP30.SHC.geni.

Figure 5 displays the structure of lyophilized hydrogels, which shows a porous structure with varied sizes. Because the freeze-drying process causes artifacts, the porosity of the dried structure does not represent the porosity of the hydrated structure. Previous research has demonstrated that altering the type and concentration of the gelling agent can result in varied morphologies, as well as affect the amount of oxygen and nutrients available to the cells, and hence cell survival (Ceccaldi et al., 2017).

Here again, the observed difference between the gels indicates that the morphology can be defined by altering the type and concentration of gelling agent. The optimal morphology is selected depending on the application of scaffold. The amount of porosity and the size of the pore (larger or smaller) can be regulated by the application of scaffold, and the amount of porosity affects cell invasion, tissue differentiation, and drug release. By comparing the hydrogel to a poreless freeze-dried chitosan film, Figure 5 shows that the concentrations of GP and SHC may be adjusted to control the pore shape in scaffolds. In comparison to the GP50 sample, the porosity of the GP30.SHC sample increased. The existence of SF polymer in the hydrogel results in a layered structure with small pores. The GP30.SHC.The SF sample has a porous structure with pore sizes ranging from a few microns to more than 100 microns, as shown in Figure 5B–E. As shown in the study by Moura et al. (2013), gels crosslinked with genipin exhibit an open network structure that appears to be quite porous with the interconnecting macrodomains.

The pore structure is a crucial consideration in tissue engineering scaffolds’ design. Cells, as well as water and biological fluids, can migrate within the interconnected porous structure of the hydrogel scaffold. Cell migration is restricted when pores are insufficiently large, leading to the creation of a cellular capsule around the borders of the scaffold. This can then lead to nutrition transport and waste removal, resulting in necrotic areas inside the construct. Overly large pores, on the other hand, reduce surface area, inhibiting cell adhesion (Murphy et al., 2010). The optimum porous structure of GP30.SHC.SF.geni and GP30.SHC.geni samples makes them useful as injectable scaffolds over a wide range of tissue engineering applications and cell types. As added benefits, these scaffolds also have desirable qualities like optimal biodegradability and sufficient mechanical strength.

#### 3.1.5 Mechanical properties

Injectable scaffolds, with sufficient mechanical characteristics, maintain integrity in the face of *in vitro* stress. This indicates that hydrogels can be employed as a matrix for cell therapy applications. The elastic constant, E, of the matrix or microenvironment, is used to calculate the resistance that a cell experiences when deforming the ECM. Gelling agents, crosslinkers, and concentrations of hydrogels’ components influence their mechanical properties and elasticity, resulting in controlled cell differentiation and tissue formation. Hydrogels also imitate the ECM and produce a microenvironment with mechanical cues closer to natural tissue (Kim et al., 2014).

Cell adhesion and growth in the hydrogel scaffold are affected by cell interaction with the surrounding microenvironment, and biochemical substances have varied impacts on cell behavior under different mechanical conditions of the microenvironment. In cell-cell and cell-matrix adhesions, actin and myosin filaments, as well as mechanical sensors that monitor the force acting on the matrix or cell, play a vital role. Through actin-myosin contractions, focal adhesions provide cells with the essential force transmission routes to “feel” their microenvironment (Engler et al., 2006). The stiffness of the scaffold can be tuned to different values regarding the target tissue and cell. The mechanical stimulation of the microenvironment by the scaffold stimulates the mechanical sensor of cells and initiates intracellular cascades, resulting in differentiated gene expression and cell function, such as proliferation, migration, or changes in how cells are arranged next to each other (Song et al., 2018).

**FIGURE 6:**
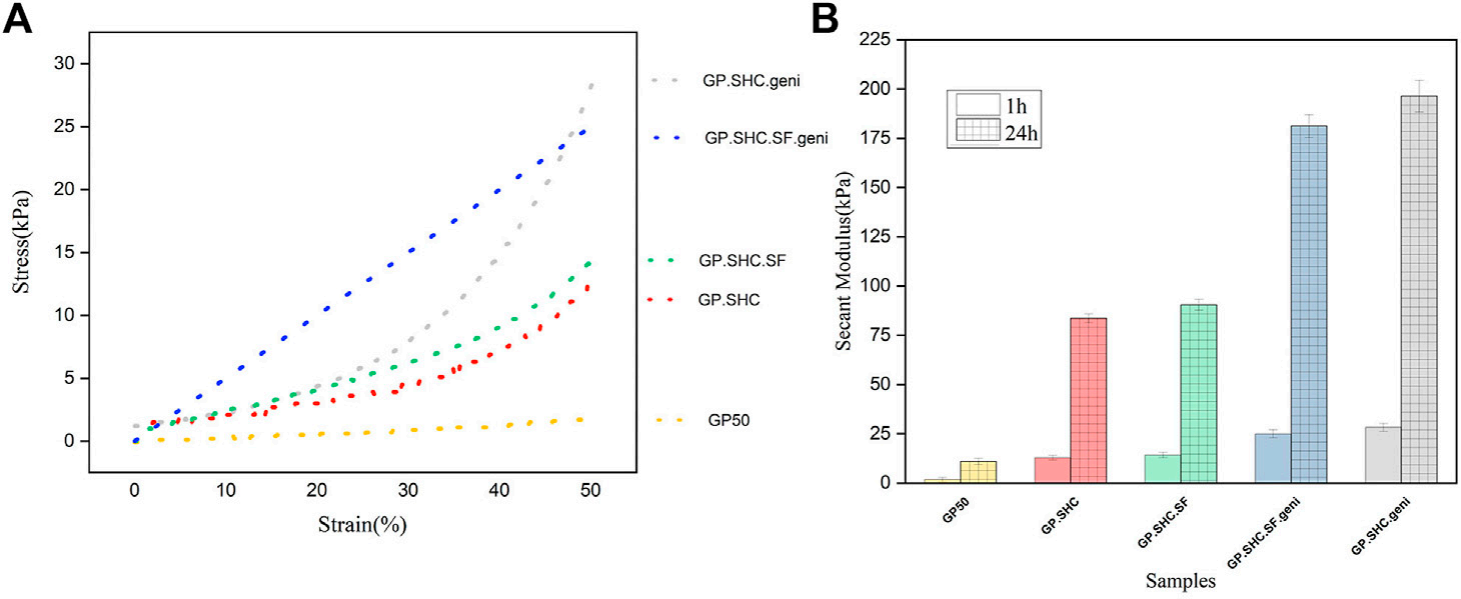
Mechanical properties of hydrogels. **(A)** Stress-strain diagram of hydrogels after 1 h of gelation and **(B)** Comparison of Secant modulus that measured at 50% of deformation after 1 and 24 h gelation, (mean + SD; *n* = 3).

Figure 6A shows stress-strain diagrams for hydrogels after 1 h of gelation. In addition, after 1 h and 24 h of gelation, Figure 6B demonstrates the Young secant moduli of hydrogels in compression (calculated at 50% deformation, showing their mechanical characteristics). The secant modulus after 24 h of gelling is more than the amount after 1 h for all specimens, indicating that the gelling procedure continues even after 1 h, particularly in genipin-containing specimens, and is consistent with Assaad et al. (2015).

In comparison to the GP50, the strain–stress curves for GP30. SHC and GP30. SHC.SF gels were relatively steep with non-linear features; hence the results are displayed in secant modulus. The secant modulus increased as the hydrogel was deformed, indicating typical non-linear behavior (Assaad et al., 2015).

The low Young modulus (5 kPa) of GP50 gel shows its softness and weakness, but the GP30.SHC gel has higher secant moduli (8 kPa at 50% deformation). When these two samples are compared, it can be concluded that the presence of SHC and GP chemicals in the structure raises the sample modulus. The complete neutralization of polymer chains, the smaller size of the SHC molecule, and its degradation into CO_2_ may explain the improved mechanical properties of SHC-containing gels compared to GP (Alinejad et al., 2018). The secant modulus increased from 8 kPa for GP50 to 14 kPa for GP30. SHC.SF after the addition of silk fibroin.

The genipin content increased the modulus of GP30.SHC.SF.geni and GP30.SHC.geni despite reduced salt concentrations compared to GP50, Figure 6B. The increased modulus of GP30.SHC.SF.geni and GP30.SHC.geni gels, in comparison with all samples, can be attributed primarily to the effects of genipin. On the other hand, when comparing these two samples, the SF component distinguishes GP30.SHC.SF from GP30.SHC.

Gelling agent salts will interact with the polar groups of SF and chitosan, which include free hydroxyl groups as well as terminated amino and carboxyl groups, to increase the strength of the hydrogels. On the other hand, silk fibroin chains can be converted into two different configurations: helixes and sheets, with the beta-sheet structure being more stiff and beneficial for reinforcing the composite or its compounds (Wu et al., 2016). Genipin crosslinker increase beta-sheet regions with crystalline domains, increasing stiffness and modulus (Silva et al., 2008; Wu et al., 2016). These findings indicate that these hydrogels could be employed as therapeutic cell delivery vehicles with desired mechanical properties.

#### 3.1.6 Swelling measurement

Capacity for water retention in the ECM, which supports many cellular activities and functions, is a significant feature, according to Amorim et al. (2021). Swelling behavior is one of the most significant aspects of hydrogel hydrophilicity for evaluating hydrogels and determining their short-term durability following *in vivo* implantation. Because channels with large pores and significant porosity within the gel can help the hydrogel swell, the swelling index of dry hydrogel can also measure pore size and porosity (Yoshida et al., 1994). Three replications of each specimen were used to determine the swelling property of the hydrogel. The swelling capacity of scaffolds is affected by several parameters, including crosslink degree, pH, temperature, and the hydrophilicity of the polymer structure.

**FIGURE 7:**
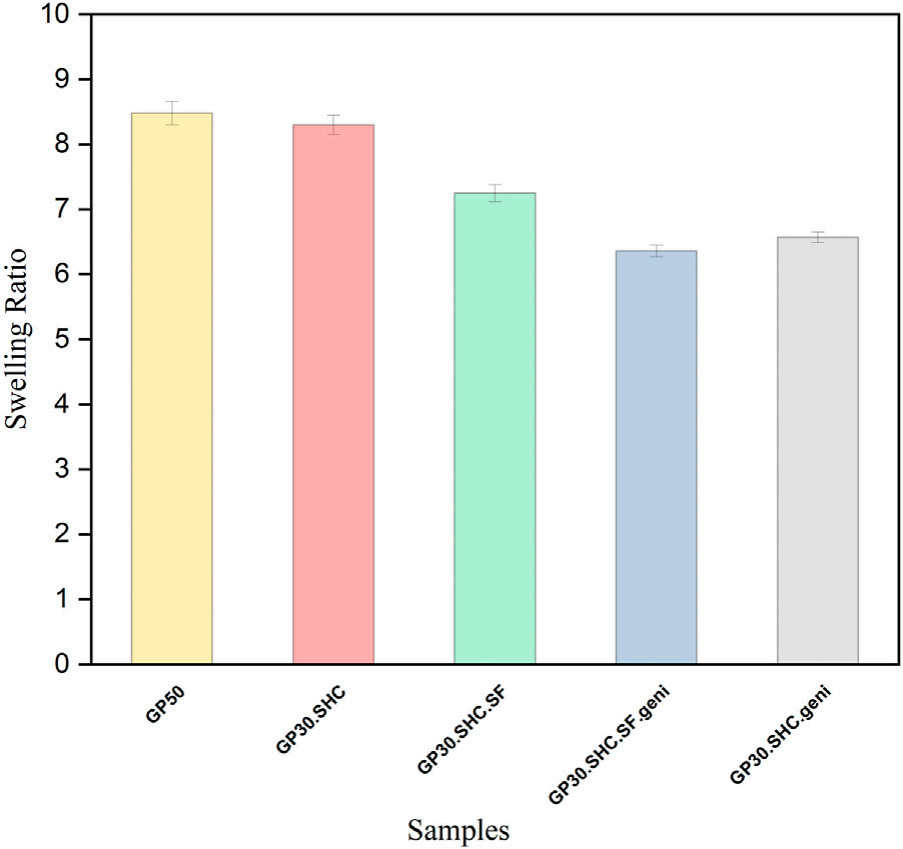
Swelling characteristicsof Hydrogel within the first 160 min of immersion in water. Error bars represent the mean ± standard deviation (*n* = 3).

The swelling ratio in PBS for gels is shown in Figure 7. Degrees of swelling are highest in GP50 and GP30.SHC samples represent values of 8.2 and 8.4, respectively. Increased crosslinking of amine groups in chitosan and silk fibroin causes a compact and stable microstructure and fewer accessible amine groups, reducing the swelling ratio by 6.2 and 6.5, respectively, in genipin-containing samples, i.e., GP30.SHC.geni and GP30.SHC.SF.geni. Song et al. (2018) reported similar results when they investigated the properties of chitosan–gelatin scaffolds crosslinked with different genipin concentrations. The swelling capacity of the genipin crosslinked scaffolds was lower than that of the similar non-crosslinked scaffold, which was related to the increased degree of crosslinking.

The interchain polymer interactions as well as the formation of hydrogen bonds, are increased in GP30.SHC.SF.geni and GP30.SHC.SF compared to the GP50 and GP30.SHC without silk fibroin. Because newly formed hydrogen bonds constrain the mobility or relaxation of network chains in hydrogels, the swelling rate is decreased when internal and external polymer chain interactions produce a stronger network. When it comes to GP30.SHC.SF.geni, the synergistic actions of polymers and crosslinkers will further limit gel swelling, resulting in a reduction in the swelling index.

#### 3.1.7 *In vitro* biodegradation study

**FIGURE 8:**
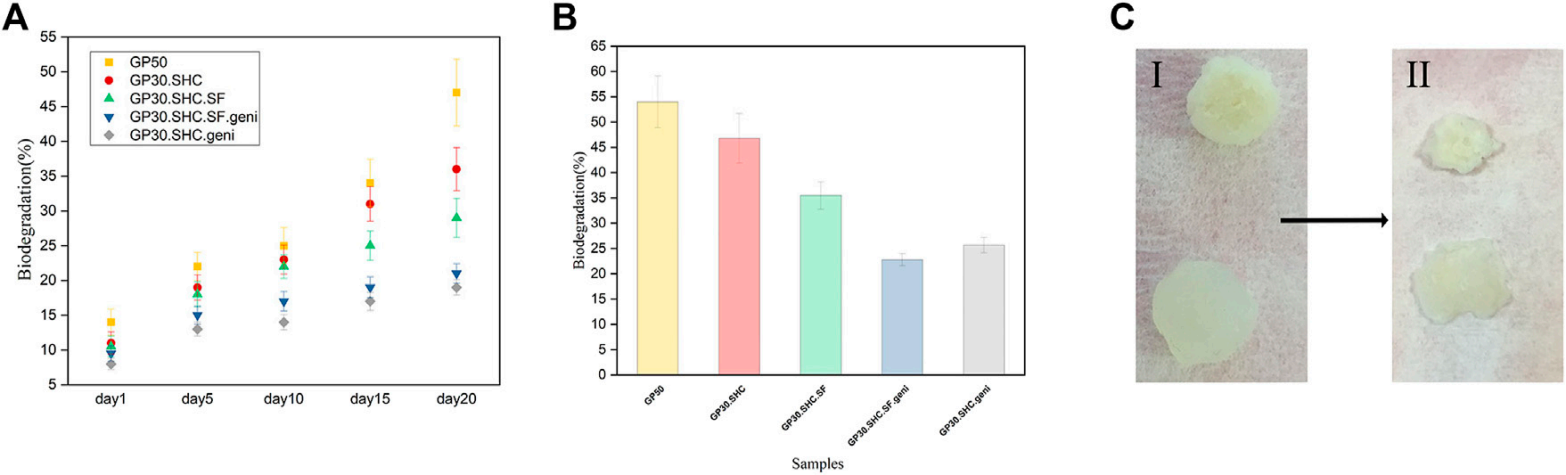
*In vitro* biodegradation of hydrogels. **(A)** biodegradation profiles of the hydrogels incubated within buffer solution at 37°C for 20 days. Error bars represent the mean ± standard deviation (*n* = 3) **(B)** biodegradation ratio of hydrogels after 28 days **(C)** hydrogels in (I) day 1 and (II) day 20.

The role of tissue engineering scaffolds after implantation is related to their resistance to biodegradation in the body; consequently, the hydrogel must be strong enough to operate correctly as a carrier for drug, bioactive molecule, or cell transport over a long period of time. However, the scaffold must degrade after the cells have been seeded onto the scaffold, proliferated, and begun to generate their ECM. Once new tissues have developed, it is crucial that the scaffold material completely degrade and be safely absorbed by the body. The rate of hydrogel deterioration was determined by monitoring the weight loss of samples while they were stored in buffer at 37°C for a period of 30 days. In order to mimic the *in vivo* degradation mechanism, the gels were immersed in a pH 7.4 buffer solution containing 1,000 units of lysozyme per milliliter and incubated at 37 C for 3 days (Li et al., 2017). The lysozyme was utilized to investigate the enzymatic degradation of the scaffold. Weight loss was faster for hydrogels without genipin, as expected. Crosslinking the hydrogel network improves the stability of the scaffold. The degradation rate of the genipin crosslinked GP30.SHC.geni and GP30.SHC.SF.geni hydrogels are reduced to 29% and 23%, respectively, as shown in Figure 8B, while the hydrogel without genipin crosslinking has a biodegradability rate of around 54%. A recent study confirmed that hydrogels crosslinked with genipin represented a decreased *in vitro* hydrogel degradation (Song et al., 2018; Zafar et al., 2021).

Chitosan and silk fibroin are natural polymers with a structure that affects their degradation rate. The deacetylation degree (DD) of Cs is related to its degradation. The Cs with a lower DD has more acetyl groups (NHCOCH_3_) and amorphous areas, which impacts the crystallinity of chitosan, resulting in faster *in vitro* degradation. Consequently, the deacetylation degree of the chitosan employed is 90%, influencing the biodegradability rate (Ahmed et al., 2018; Bakshi et al., 2020).

The binding of N-acetylglucosamine residues to the active site of lysozyme has been suggested as a reason for the degradation of chitosan by lysozyme. Horan’s team (Horan et al., 2005) placed SF in PBS to monitor the degradation process and discovered that the SF mass remained constant after 10 days. In the current investigation, CS degradation likely contributed to the rapid initial rate of scaffold material degradation.

For silk-containing hydrogels to be effective as scaffolds that can withstand enzymatic degradation, the protein must undergo a conformational change from random coils to very stable sheets. Crystal-sheet conformation of repetitive amino acid sequences makes up the hydrophobic domain of silk polymeric chains, and the hydrophilic links between these hydrophobic domains consist of bulky and polar side chains that form the amorphous random coil conformation, as previously mentioned (Horan et al., 2005).

The weight of degraded gel samples was measured, and the results are shown in Figure 8A. It can be observed that 1) the GP50 gel lost a great deal of its weight after a 3-week degradation period, with a degradation ratio of roughly 46%, and 2) starting from day 10, the GP30.SHC.SF and GP30.SHC gels lost weight much slower than the GP50. The comparison in Figure 8A suggests that the silk fibroin content in GP30.SHC.SF and GP30.SHC.SF.geni hydrogels is effective in slowing down the degradation process since silk fibroin degrades more slowly than chitosan (Bhattarai et al., 2010). Intermolecular interactions are greater in these samples during gel formation, enhancing the strength of gel and postponing the degradation of the scaffold.

The size of the hydrogel specimens diminishes during the biodegradation process, as shown in Figure 8C, and the scaffold appears to have deteriorated through surface and interior erosion.

Finally, each of these scaffolds can be chosen for a specific application depending on the injection site of the target in the body and the amount of time required for the gel to remain in the body. However, genipin-containing scaffolds, namely GP30.SHC.SF.geni and GP30.SHC.geni, are the optimal solution in these hydrogels, with higher strength and degradation tolerance as well as improved injection performance.

### 3.2 Biological evaluation

#### 3.2.1 MTT assay

**FIGURE 9:**
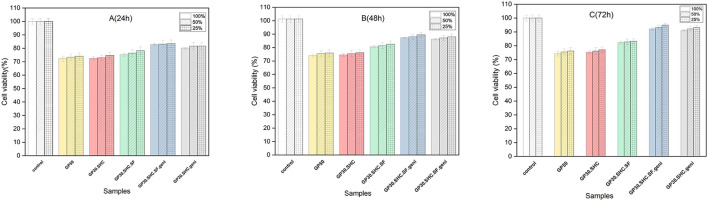
The MTT findings after exposing fibroblasts for **(A)** 24, **(B)** 48, and **(C)** 72 h to different dilutions of hydrogels’ extraction medium (25, 50, 100%).

The viability of fibroblast cells was assessed using MTT assays in varied concentrations of extraction media (25, 50, 100%) after 24, 48, and 72 h of incubation to assess the cytotoxicity of hydrogels using the extract dilution method. As predicted from the biodegradation data, day 1 of cell culture in GP50 resulted in decreased cell viability Figure 9. According to prior research, chitosan hydrogel produced at a concentration of 0.2 M GP or less exhibited no cytotoxic effects; non-etheless, its sluggish gelling rate limited its applicability in a variety of contexts. Hydrogels made from chitosan with GP concentrations high enough to induce quick gelation (0.4 M GP or more) caused considerable cell death due to the rapid release of a large quantity of GP upon immersion in the culture medium, leading to hypertonicity of the media extracts (Ahmadi and de Bruijn, 2008).

Indirect cytotoxicity testing revealed that these new hydrogel formulations were more cytocompatible than GP50 hydrogel. No cytotoxic effect was seen in this investigation when extracts from the new formulations were used. This may be due to the fact that this innovative gelling agent system used to create the hydrogel lowers the total salt content of the hydrogel, saving cells from hyperosmolality that would otherwise damage or kill them. Furthermore, because SHC is an integral component of the blood chemical buffer system, it is biocompatible at low concentrations. SHC 0.1 M caused no damage to endothelial cells in previous studies (Hirsch and Haller, 2004), but it had a significant cytotoxic effect at high concentrations (2.4 M) (Assaad et al., 2015).

Chitosan structural units are structurally similar to glycosaminoglycans, making them biocompatible. They interact with growth factors, receptors, and adhesive proteins in cells. Thus, chitosan is believed to have a biocompatible structure for cells.

In 24 h, the viability of GP30.SHC.SF and GP30.SHC.SF.geni containing silk-fibroin polymer was higher (75% and 82%, respectively), and the number of dead cells was reduced. The biocompatibility properties of silk fibroin are determined by the conformation and structure of its amino acids. The formation of the beta-sheet structure increases the biocompatibility of silk fibroin scaffolds, confirming the findings of earlier studies (Yang et al., 2019; El-Fakharany et al., 2020). Silk fibroin has recently been discovered to diminish reactive oxygen species (ROS) created by activated neutrophils and macrophages (Yang et al., 2019; El-Fakharany et al., 2020). These ROS then stimulate the synthesis of a variety of non-specific mediators, including histamine, serotonin, and interleukin, which in turn attract even more inflammatory cells. Routine processes of the body, which include proteolytic enzymes and free radical scavenger chemicals, neutralize ROS and its byproducts (Kundu et al., 2013; Ezhilarasi et al., 2020).

*In vitro* biocompatibility of scaffolds containing genipin was excellent. In 24 h, the cell viability of the GP30.SHC.SF.geni and GP30.SHC.geni samples were about 82% and 80%, respectively, and increased to 91% and 89% in 72 h. All formulations significantly increased fibroblast vitality after 72 h, which may be attributable to the cells’ adaptability *via* the release of growth factors, as mentioned in a previous study by Ahmadi and de Bruijn (2008).

The presence of genipin produces crosslinking in the hydrogel structure, which inhibits hydrogel biodegradation and leads to lower extract concentrations, which may reduce cytotoxicity. The cell viability was 72%–91% with genipin crosslinking, as shown in Figure 9 (Ye et al., 2019), demonstrating that the hydrogels had low cytotoxicity.

Two samples of injectable chitosan hydrogel with this novel formulation, GP30.SHC.SF.geni and GP30.SHC.geni, which incorporates polymers with a gelling agents-crosslinking system, were found to be promising carriers that did not compromise cell viability *in vitro* cytotoxicity tests and other assays. Thus, these two formulations were selected for 3D cell culture and urea testing.

#### 3.2.2 Hemolysis

**FIGURE 10:**
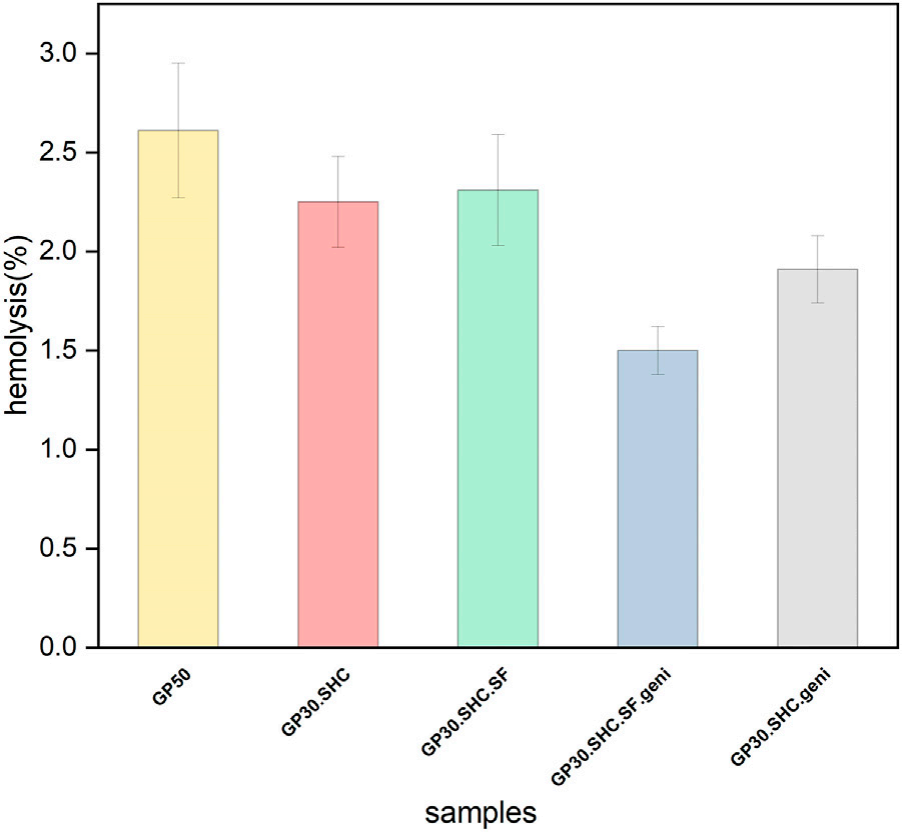
Hemolysis rate of scaffold samples.

It is permissible to use *in vitro* hemolysis as a credible indicator method for determining the hemocompatibility of the scaffold. It is used as an indication of damage to the membrane of red blood cells. The hemolysis value is considered safe when it is below 5%, in accordance with the standard ISO document 10993-5 1992 (Seibert et al., 2003). According to the experimental approach, the scaffolds examined in this test were safe, and the hemolytic activity for all specimens was less than 5%, as shown in Figure 10. The lack of hemolytic properties of the chitosan-glycerophosphate scaffold was also confirmed in previous investigations by Zhou et al. (2011).

In the sol-gel phase transition, we have a wide range of molecular interactions between the aqueous solution of cationic chitosan and the anionic salt bases of the gelling agents, as discussed in the preceding sections. These interactions appear to decrease cationic density of chitosan, affecting hemolytic properties of the hydrogel structure. In reality, the hemolytic response is influenced by the interactions of the chitosan-related positive amine group with plasma proteins and blood cells. It appears that a hemocompatible polymer, as described by Ostuni et al. (2001), should be a hydrophilic material that is electrically neutral and has hydrogen-bond acceptors. When a polymer or scaffold comes into direct interaction with blood, it triggers a cascade of host responses. Protein adsorption to the surface, neutrophil and macrophage activation, coagulation cascade activation, platelet adhesion, complement system activation, antibody production, and cellular immune response are all related to these responses. These responses must be minimized or avoided by a hemocompatible system (Balan and Verestiuc, 2014).

Some polymers have the potential to break red blood cell membranes and release hemoglobin and other internal constituents after being exposed to them. The release of these components speeds up platelet accumulation and clot formation in that area. After the material is exposed to the blood, the protein adsorption on the interface of the material is the first event that occurs. Another group of proteins substitutes the absorbed proteins in the second step, irreversible changes in their conformation arise, and active platelet receptors detect the layer in which the protein is absorbed. Platelet adhesion and the clotting process are examples of subsequent reactions. Surface charge density of the material critically impacts the system (Balan and Verestiuc, 2014; Ashcraft et al., 2021).

Chemical composition of the associated material, structure, and topography can also influence protein adsorption (Nonckreman et al., 2010). Chitosan and silk fibers are positively charged polymers of the hydrogel system; however, these hydrogels are created using glycerophosphate and sodium hydrogen carbonate gelling agents, as well as positive linkages between polymer constituents. It efficiently reduces the contact between blood cells and the material, hence the reaction of activated blood cells to the scaffold.

The presence of genipin, on the other hand, causes crosslinking in the structure, which reduces the size of the pores and reduces initial protein absorption. As a result, in hydrogels containing genipin, the hemolysis ratio decreases, and thus, the rate of hemocompatibility increases. The hemolysis test findings suggest that this chitosan-based injectable hydrogel system is safe and also has a modest hemolytic effect.

#### 3.2.3 Cell viability assessment in hydrogels

**FIGURE 11:**
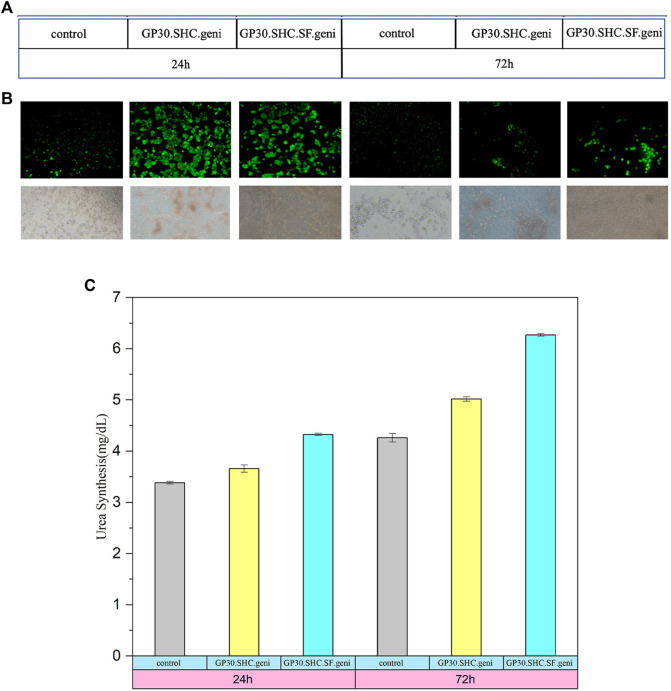
Evaluation of the HepG2 cells cultured in the hydrogels. **(A)** HepG2 cells encapsulated at day 1 and day 3. The cells were stained with FDA/PI (green) and (red) dyes, respectively. **(B)** encapsulated HepG2 cells on day 1 and day 3 visualized by optical microscopy. Scale bars: 200 μm **(C)** urea synthesis in the encapsulated HepG2 cell line. Secretion in 2D culture as control. Data represent value ± SD from three samples.

The HepG2 cell line was encapsulated in two of the best hydrogel formulations previously selected (GP30.SHC.SF.geni and GP30.SHC.geni). The metabolic activity of the cells (urea synthesis) was also monitored for 24 and 72 h, as represented in Figure 11C, and live/dead labeling was done after 24 h of culture Figure 11A. The staining of living cells with fluorescein diacetate (FDA) causes them to fluoresce green following the response of intracellular esterase, whereas the staining of dead cells with propidium iodide (PI) results in a red coloration (Boyd et al., 2008).

Encapsulated cells are intended to be viable and functioning for implantation throughout the body. The quantification of live/dead cells by image analyses showed good cell survival and no difference between the two formulations evaluated, as shown in Figure 11A, B. After 24 h in culture, the majority of cells were alive (cells in green), and just a small percentage had died (cells in red). The survival of encapsulated cells is influenced by the physiological pH of hydrogel and the existence of interconnecting pores inside the hydrogel, providing access to nutrients and oxygen (Ceccaldi et al., 2017; Hori et al., 2021). We also hypothesized that biodegradability of hydrogel would offer a nutrient-rich, favorable environment for the encapsulated cells, leading to increased cell viability. These three-dimensional hydrogel scaffolds, we hypothesized, would support significant cell-cell communication, leading to improved hepatocyte viability and functionality.

According to Capone et al. (2013), Hori et al. (2021), seeded cells have a spindle-like shape; however, encapsulated cells have a round morphology inside the hydrogel. The spherical shape of the encapsulated cells in the 3D gel suggests that the polymer chains did not adhere to the cells, which means that the cells were able to survive during the culture time. Finally, HepG2 cells encapsulated during the crosslinking procedure to create cell-hydrogel structures prove to be advantageous for cell survival and efficiency, which can be employed for cell therapy and tissue engineering applications. The same results for other formulations have been obtained by Lan et al.( 2010), Hori et al, (2021)

#### 3.2.4 Urea synthesis

One of the most crucial factors of hepatocyte health and function is urea secretion. The examination of a liver metabolic function will be the following step. On days 1 and 3, urea synthesis was investigated in supernatants to assess the hepatic functionality of beads. As a control, urea synthesis was measured in cells grown in collagen-coated 24-well plates.

Due to its importance in ammonia detoxication and pH control, ammonia metabolism is often used as a functional marker of hepatic phenotype (Godoy et al., 2013). The urea cycle, which converts extremely toxic ammonia to urea for excretion, is primarily carried out in the liver. As a result, urea production levels in blood or culture supernatant indicate liver functionality. Low urea levels, in particular, suggest liver dysfunction (Kang et al., 2021).

As shown in Figure 11, improved liver-specific functions are directly linked to increased cell viability in the encapsulation culture. Figure 11C demonstrates that the urea level under the encapsulation condition is much greater at each time point than HepG2 cultured alone, which could be due to cell-matrix interactions, which can increase liver-specific functions (Cui et al., 2019).

Between GP30.SHC.SF.geni and GP30.SHC.geni, no significant differences were detected. The difference between day 1 and day 3 was, however, more apparent than in the experimental groups. The synthesis of urea in GP30.SHC.SF.geni and GP30.SHC.geni was considerably higher on day 3 (6.269 ± 0.023 and 5.017 ± 0.042 mg dl^−1^, respectively) than on day 1 (4.326 ± 0.021 and 3.659 ± 0.071 mg dl^−1^).

Hepatocytes cultured in different 3D scaffolds of other studies showed similar enhancements in liver-specific activities (Wu et al., 2014; Wang et al., 2019). The improved microencapsulated cell viability outcomes are consistent with the liver-specific function data, suggesting the benefits of the 3D culture system (Khodabakhshaghdam et al., 2021). This indicates that the 3D culture system with a specific hydrogel combination had no cytotoxic effect on cell metabolic activity. Overall, our findings indicate that the 3D hydrogel provides an alternate method of cell growth, resulting in the formation of a functional microtissue *in vitro*.

## 4 Conclusion

Hydrogel scaffolds that rapidly solidify at body temperature have raised great interest in cell therapy and tissue engineering purposes. Previous studies demonstrate that chemical crosslinkers, compared to physical ones, result in strong mechanical properties of biological scaffolding systems. At the same time, due to the necessity for high-concentration usage, they induce increased cytotoxicity. In order to mimic *in vivo* situations, we designed and evaluated new *in-situ* gelling chitosan/silk fibroin hydrogels using the novel gelling agent system consisting of GP and SHC, crosslinked with genipin. These hydrogels, formulated using natural ingredients, are highly biocompatible (72%–85%) and sufficiently biodegradable (about 23% after 28 days), all allow the production of *in-situ* crosslinking systems with pH adjustments (range of natural pH). Furthermore, the thermoresponsive features of these hydrogels enable homogeneous mixing with cells and rapid gelling at 37°C (230 s), preventing cells from spreading out of the targeted area and maintaining matrix cohesiveness due to strong ultimate mechanical strength (185 kPa after 24 h of gelation). The stronger intermolecular interactions brought about by the novel gell agent-crosslinker system are responsible for the enhanced mechanical properties and prolonged degradation of the scaffold. Improved hemocompatibility and lower hemolysis ratios are the outcomes of genipin presence in crosslinked structures *via* decreasing pore size and delaying early protein absorption. By evaluating urea production, the positive effect of cell-matrix interactions and mimicking the extracellular matrix on the improvement of viability and, thus, liver-specific activities of encapsulated cells have been clearly observed. Genipin crosslinked hydrogels showed tremendous promise for use in 3D cell cultivation, with urea production increasing from 4 mg dl^−1^ on day 1 to roughly 6 mg dl^−1^ on day 3. The setting pH of hydrogel, close to the physiological one, and their interconnected pores, providing access to nutrients and oxygen, could describe the survival of encapsulated cells. High-throughput, minimally invasive surgery may have a promising future thanks to these kinds of injectable, thermoresponsive hydrogels.

## Data Availability

The original contributions presented in the study are included in the article/Supplementary Material, further inquiries can be directed to the corresponding author.

## References

[B1] Afewerki S. Sheikhi A. Kannan S. Ahadian S. Khademhosseini A. (2019). Gelatin‐polysaccharide composite scaffolds for 3D cell culture and tissue engineering: towards natural therapeutics. Bioeng. Transl. Med. 4 (1), 96–115. 10.1002/btm2.10124 30680322 PMC6336672

[B2] Ahmadi R. de Bruijn J. D. (2008). Biocompatibility and gelation of chitosan–glycerol phosphate hydrogels. J. Biomed. Mater. Res. Part A 86 (3), 824–832. 10.1002/jbm.a.31676 18041728

[B3] Ahmed S. Ali A. Sheikh J. (2018). A review on chitosan centred scaffolds and their applications in tissue engineering. Int. J. Biol. Macromol. 116, 849–862. 10.1016/j.ijbiomac.2018.04.176 29730001

[B4] Alinejad Y. Adoungotchodo A. Hui E. Zehtabi F. Lerouge S. (2018). An injectable chitosan/chondroitin sulfate hydrogel with tunable mechanical properties for cell therapy/tissue engineering. Int. J. Biol. Macromol. 113, 132–141. 10.1016/j.ijbiomac.2018.02.069 29452185

[B5] Amorim S. Reis C. A. Reis R. L. Pires R. A. (2021). Extracellular matrix mimics using hyaluronan-based biomaterials. Trends Biotechnol. 39 (1), 90–104. 10.1016/j.tibtech.2020.06.003 32654775

[B6] Ardeshirylajimi A. Delgoshaie M. Mirzaei S. Khojasteh A. (2018). Different porosities of chitosan can influence the osteogenic differentiation potential of stem cells. J. Cell. Biochem. 119 (1), 625–633. 10.1002/jcb.26223 28618050

[B7] Ashammakhi N. Ahadian S. Darabi M. A. El Tahchi M. Lee J. Suthiwanich K. (2019). Minimally invasive and regenerative therapeutics. Adv. Mater. 31 (1), 1804041. 10.1002/adma.201804041 PMC6709364 30565732

[B8] Ashcraft M. Douglass M. Chen Y. Handa H. (2021). Combination strategies for antithrombotic biomaterials: An emerging trend towards hemocompatibility. Biomater. Sci. 9 (7), 2413–2423. 10.1039/d0bm02154g 33599226 PMC8035307

[B9] Assaad E. Maire M. Lerouge S. (2015). Injectable thermosensitive chitosan hydrogels with controlled gelation kinetics and enhanced mechanical resistance. Carbohydr. Polym. 130, 87–96. 10.1016/j.carbpol.2015.04.063 26076604

[B10] Bakshi P. S. Selvakumar D. Kadirvelu K. Kumar N. (2020). Chitosan as an environment friendly biomaterial–a review on recent modifications and applications. Int. J. Biol. Macromol. 150, 1072–1083. 10.1016/j.ijbiomac.2019.10.113 31739057

[B11] Balan V. Verestiuc L. (2014). Strategies to improve chitosan hemocompatibility: A review. Eur. Polym. J. 53, 171–188. 10.1016/j.eurpolymj.2014.01.033

[B12] Bhattarai N. Gunn J. Zhang M. (2010). Chitosan-based hydrogels for controlled, localized drug delivery. Adv. drug Deliv. Rev. 62 (1), 83–99. 10.1016/j.addr.2009.07.019 19799949

[B13] Boyd V. Cholewa O. M. Papas K. K. (2008). Limitations in the use of fluorescein diacetate/propidium iodide (FDA/PI) and cell permeable nucleic acid stains for viability measurements of isolated islets of Langerhans. Curr. trends Biotechnol. Pharm. 2 (2), 66–84. 20814586 PMC2931281

[B14] Brouwer J. van Leeuwen-Herberts T. Otting-van de Ruit M. (1984). Determination of lysozyme in serum, urine, cerebrospinal fluid and feces by enzyme immunoassay. Clin. Chim. Acta 142 (1), 21–30. 10.1016/0009-8981(84)90097-4 6383662

[B15] Butler M. F. Ng Y. F. Pudney P. D. (2003). Mechanism and kinetics of the crosslinking reaction between biopolymers containing primary amine groups and genipin. J. Polym. Sci. Part A Polym. Chem. 41 (24), 3941–3953. 10.1002/pola.10960

[B16] Capone S. H. Dufresne M. Rechel M. Fleury M-J. Salsac A-V. Paullier P. (2013). Impact of alginate composition: from bead mechanical properties to encapsulated HepG2/C3A cell activities for *in vivo* implantation. PLoS One 8 (4), e62032. 10.1371/journal.pone.0062032 23637958 PMC3636232

[B17] Cárdena-Pérez Y. C. Vera-Graziano R. Muñoz-Prieto EdJ. Gómez-Pachón E. Y. (2017). Preparation and characterization of scaffold nanofibers by electrospinning, based on chitosan and fibroin from Silkworm (*Bombyx mori*). Ing. Compet. 19 (1), 139–151.

[B18] Ceccaldi C. Assaad E. Hui E. Buccionyte M. Adoungotchodo A. Lerouge S. (2017). Optimization of injectable thermosensitive scaffolds with enhanced mechanical properties for cell therapy. Macromol. Biosci. 17 (6), 1600435. 10.1002/mabi.201600435 28116831

[B19] Celikkin N. Rinoldi C. Costantini M. Trombetta M. Rainer A. Święszkowski W. (2017). Naturally derived proteins and glycosaminoglycan scaffolds for tissue engineering applications. Mater. Sci. Eng. C 78, 1277–1299. 10.1016/j.msec.2017.04.016 28575966

[B20] Chenite A. Chaput C. Wang D. Combes C. Buschmann M. Hoemann C. (2000). Novel injectable neutral solutions of chitosan form biodegradable gels *in situ* . Biomaterials 21 (21), 2155–2161. 10.1016/s0142-9612(00)00116-2 10985488

[B21] Cho J. Heuzey M-C. Bégin A. Carreau P. J. (2005). Physical gelation of chitosan in the presence of β-glycerophosphate: the effect of temperature. Biomacromolecules 6 (6), 3267–3275. 10.1021/bm050313s 16283755

[B22] Cui J. Wang H. Shi Q. Sun T. Huang Q. Fukuda T. (2019). Multicellular co-culture in three-dimensional gelatin methacryloyl hydrogels for liver tissue engineering. Molecules 24 (9), 1762. 10.3390/molecules24091762 31067670 PMC6539120

[B23] De Pieri A. Rochev Y. Zeugolis D. I. (2021). Scaffold-free cell-based tissue engineering therapies: advances, shortfalls and forecast. NPJ Regen. Med. 6 (1), 18–15. 10.1038/s41536-021-00133-3 33782415 PMC8007731

[B24] De Souza R. Zahedi P. Allen C. J. Piquette-Miller M. (2009). Biocompatibility of injectable chitosan–phospholipid implant systems. Biomaterials 30 (23-24), 3818–3824. 10.1016/j.biomaterials.2009.04.003 19394688

[B25] El-Fakharany E. M. Abu-Elreesh G. M. Kamoun E. A. Zaki S. Abd-El-Haleem D. A. (2020). *In vitro* assessment of the bioactivities of sericin protein extracted from a bacterial silk-like biopolymer. RSC Adv. 10 (9), 5098–5107. 10.1039/c9ra09419a 35498316 PMC9049123

[B26] Engler A. J. Sen S. Sweeney H. L. Discher D. E. (2006). Matrix elasticity directs stem cell lineage specification. Cell 126 (4), 677–689. 10.1016/j.cell.2006.06.044 16923388

[B27] Ezhilarasi S. S. V. Kothandaraman R. Nesamani R. Balasubramanian S. Mahalaxmi S. (2020). *In vitro* assessment of cytotoxicity and anti-inflammatory properties of shilajit nutraceutical: A preliminary study. J. Interdiscip. Dent. 10 (1), 24. 10.4103/jid.jid_2_20

[B28] Filion D. Lavertu M. Buschmann M. D. (2007). Ionization and solubility of chitosan solutions related to thermosensitive chitosan/glycerol-phosphate systems. Biomacromolecules 8 (10), 3224–3234. 10.1021/bm700520m 17850110

[B29] Floren M. Migliaresi C. Motta A. (2016). Processing techniques and applications of silk hydrogels in bioengineering. J. Funct. Biomater. 7 (3), 26. 10.3390/jfb7030026 27649251 PMC5040999

[B30] Godoy P. Hewitt N. J. Albrecht U. Andersen M. E. Ansari N. Bhattacharya S. (2013). Recent advances in 2D and 3D *in vitro* systems using primary hepatocytes, alternative hepatocyte sources and non-parenchymal liver cells and their use in investigating mechanisms of hepatotoxicity, cell signaling and ADME. Archives Toxicol. 87 (8), 1315–1530. 10.1007/s00204-013-1078-5 PMC3753504 23974980

[B31] Hashemi S. S. Pourfath M. R. Derakhshanfar A. Behzad-Behbahani A. Moayedi J. (2020). The role of labeled cell therapy with and without scaffold in early excision burn wounds in a rat animal model. Iran. J. Basic Med. Sci. 23 (5), 673–679. 10.22038/ijbms.2020.34324.8156 32742606 PMC7374999

[B32] Hirsch C. Haller C. (2004). Effect of extracellular hypertonicity and alkalosis on endothelial-derived EA. hy 926 cells *in vitro* . Eur. J. Med. Res. 9 (2), 71–77. 15090292

[B33] Horan R. L. Antle K. Collette A. L. Wang Y. Huang J. Moreau J. E. (2005). *In vitro* degradation of silk fibroin. Biomaterials 26 (17), 3385–3393. 10.1016/j.biomaterials.2004.09.020 15621227

[B34] Hori A. Utoh R. Yamada M. Seki M. (2021). “Preparation of microporous hydrogel sponges for 3D perfusion culture of mammalian cells,” in MATEC Web of Conferences, Sapporo, Hokkaido, Japan, September 27, 2019. (EDP Sciences). 10.1051/matecconf/202133307004

[B35] Huang Z. Yu B. Feng Q. Li S. (2011). Modification of an injectable chitosan scaffold by blending with NaHCO3 to improve cytocompatibility. Polym. Polym. Compos. 19 (9), 781–788. 10.1177/096739111101900908

[B36] Iansante V. Dhawan A. Masmoudi F. Lee C. A. Fernandez-Dacosta R. Walker S. (2018). A new high throughput screening platform for cell encapsulation in alginate hydrogel shows improved hepatocyte functions by mesenchymal stromal cells co-encapsulation. Front. Med. 5, 216. 10.3389/fmed.2018.00216 PMC6095031 30140676

[B37] Ijima H. Nakamura S. Bual R. P. Yoshida K. (2019). Liver-specific extracellular matrix hydrogel promotes liver-specific functions of hepatocytes *in vitro* and survival of transplanted hepatocytes *in vivo* . J. Biosci. Bioeng. 128 (3), 365–372. 10.1016/j.jbiosc.2019.02.014 30935781

[B38] Islam M. M. Shahruzzaman M. Biswas S. Sakib M. N. Rashid T. U. (2020). Chitosan based bioactive materials in tissue engineering applications-A review. Bioact. Mater. 5 (1), 164–183. 10.1016/j.bioactmat.2020.01.012 32083230 PMC7016353

[B39] Janani G. Nandi S. K. Mandal B. B. (2018). Functional hepatocyte clusters on bioactive blend silk matrices towards generating bioartificial liver constructs. Acta biomater. 67, 167–182. 10.1016/j.actbio.2017.11.053 29223705

[B40] Jiang T. Singh B. Choi Y-J. Akaike T. Cho C-S. (2015). “Liver tissue engineering using functional marine biomaterials,” in Functional marine biomaterials (Elsevier), 91–106.

[B41] Kane M. A. Kasper C. E. Kalinich J. F. (2009). Protocol for the assessment of potential health effects FromEmbedded metal fragments. Mil. Med. 174 (3), 265–269. 10.7205/milmed-d-02-2808 19354090

[B42] Kang H. K. Sarsenova M. Kim D-H. Kim M. S. Lee J. Y. Sung E-A. (2021). Establishing a 3D *in vitro* hepatic model mimicking physiologically relevant to *in vivo* state. Cells 10 (5), 1268. 10.3390/cells10051268 34065411 PMC8161177

[B43] Khodabakhshaghdam S. Khoshfetrat A. B. Rahbarghazi R. (2021). Alginate-chitosan core-shell microcapsule cultures of hepatic cells in a small scale stirred bioreactor: Impact of shear forces and microcapsule core composition. J. Biol. Eng. 15 (1), 14–12. 10.1186/s13036-021-00265-6 33865460 PMC8052835

[B44] Kim J. K. Kim H. J. Chung J-Y. Lee J-H. Young S-B. Kim Y-H. (2014). Natural and synthetic biomaterials for controlled drug delivery. Archives pharmacal Res. 37 (1), 60–68. 10.1007/s12272-013-0280-6 24197492

[B45] Kundu B. Rajkhowa R. Kundu S. C. Wang X. (2013). Silk fibroin biomaterials for tissue regenerations. Adv. drug Deliv. Rev. 65 (4), 457–470. 10.1016/j.addr.2012.09.043 23137786

[B46] Kwon Y-S. Lim E-S. Kim H-M. Hwang Y-C. Lee K-W. Min K-S. (2015). Genipin, a cross-linking agent, promotes odontogenic differentiation of human dental pulp cells. J. Endod. 41 (4), 501–507. 10.1016/j.joen.2014.12.002 25637194

[B47] Lan S-F. Safiejko-Mroczka B. Starly B. (2010). Long-term cultivation of HepG2 liver cells encapsulated in alginate hydrogels: a study of cell viability, morphology and drug metabolism. Toxicol. Vitro 24 (4), 1314–1323. 10.1016/j.tiv.2010.02.015 20171269

[B48] Lee H. J. Ahn J. Jung C. R. Jeung Y. J. Cho H. S. Son M. J. (2020). Optimization of 3D hydrogel microenvironment for enhanced hepatic functionality of primary human hepatocytes. Biotechnol. Bioeng. 117 (6), 1864–1876. 10.1002/bit.27328 32162676

[B49] Li D-W. Lei X. He F-L. He J. Liu Y-L. Ye Y-J. (2017). Silk fibroin/chitosan scaffold with tunable properties and low inflammatory response assists the differentiation of bone marrow mesenchymal stem cells. Int. J. Biol. Macromol. 105, 584–597. 10.1016/j.ijbiomac.2017.07.080 28802849

[B50] Li D-W. He J. He F-L. Liu Y-L. Liu Y-Y. Ye Y-J. (2018). Silk fibroin/chitosan thin film promotes osteogenic and adipogenic differentiation of rat bone marrow-derived mesenchymal stem cells. J. Biomater. Appl. 32 (9), 1164–1173. 10.1177/0885328218757767 29471713

[B51] Liao Y. He Q. Zhou F. Zhang J. Liang R. Yao X. (2020). Current intelligent injectable hydrogels for *in situ* articular cartilage regeneration. Polym. Rev. 60 (2), 203–225. 10.1080/15583724.2019.1683028

[B52] Marquardt L. M. Heilshorn S. C. (2016). Design of injectable materials to improve stem cell transplantation. Curr. stem Cell Rep. 2 (3), 207–220. 10.1007/s40778-016-0058-0 28868235 PMC5576562

[B53] Mirzaei E. Faridi-Majidi R. Shokrgozar M. A. Asghari Paskiabi F. (2014). Genipin cross-linked electrospun chitosan-based nanofibrous mat as tissue engineering scaffold. Nanomed. J. 1 (3), 137–146.

[B54] Moura M. Gil M. Figueiredo M. (2013). Delivery of cisplatin from thermosensitive co-cross-linked chitosan hydrogels. Eur. Polym. J. 49 (9), 2504–2510. 10.1016/j.eurpolymj.2013.02.032

[B55] Murphy C. M. Haugh M. G. O'brien F. J. (2010). The effect of mean pore size on cell attachment, proliferation and migration in collagen–glycosaminoglycan scaffolds for bone tissue engineering. Biomaterials 31 (3), 461–466. 10.1016/j.biomaterials.2009.09.063 19819008

[B56] Neri-Numa I. A. Pessoa M. G. Paulino B. N. Pastore G. M. (2017). Genipin: A natural blue pigment for food and health purposes. Trends food Sci. Technol. 67, 271–279. 10.1016/j.tifs.2017.06.018

[B57] Nilsen-Nygaard J. Strand S. P. Vårum K. M. Draget K. I. Nordgård C. T. (2015). Chitosan: Gels and interfacial properties. Polymers 7 (3), 552–579. 10.3390/polym7030552

[B58] Nonckreman C. J. Fleith S. Rouxhet P. G. Dupont-Gillain C. C. (2010). Competitive adsorption of fibrinogen and albumin and blood platelet adhesion on surfaces modified with nanoparticles and/or PEO. Colloids Surfaces B Biointerfaces. 77 (2), 139–149. 10.1016/j.colsurfb.2010.01.014 20171850

[B59] Ostuni E. Chapman R. G. Holmlin R. E. Takayama S. Whitesides G. M. (2001). A survey of structure− property relationships of surfaces that resist the adsorption of protein. Langmuir 17 (18), 5605–5620. 10.1021/la010384m

[B60] Panjapheree K. Kamonmattayakul S. Meesane J. (2018). Biphasic scaffolds of silk fibroin film affixed to silk fibroin/chitosan sponge based on surgical design for cartilage defect in osteoarthritis. Mater. Des. 141, 323–332. 10.1016/j.matdes.2018.01.006

[B61] Pankongadisak P. Suwantong O. (2019). Enhanced properties of injectable chitosan-based thermogelling hydrogels by silk fibroin and longan seed extract for bone tissue engineering. Int. J. Biol. Macromol. 138, 412–424. 10.1016/j.ijbiomac.2019.07.100 31323268

[B62] Pettinelli N. Rodriguez-Llamazares S. Bouza R. Barral L. Feijoo-Bandin S. Lago F. (2020). Carrageenan-based physically crosslinked injectable hydrogel for wound healing and tissue repairing applications. Int. J. Pharm. 589, 119828. 10.1016/j.ijpharm.2020.119828 32871220

[B63] Rijal N. Adhikari U. Bhattarai N. (2017). “Production of electrospun chitosan for biomedical applications,” in Chitosan based biomaterials (Elsevier), Vol. 1, 211–237.

[B64] Rinaudo M. Pavlov G. Desbrieres J. (1999). Influence of acetic acid concentration on the solubilization of chitosan. Polymer 40 (25), 7029–7032. 10.1016/s0032-3861(99)00056-7

[B65] Saheli M. Sepantafar M. Pournasr B. Farzaneh Z. Vosough M. Piryaei A. (2018). Three‐dimensional liver‐derived extracellular matrix hydrogel promotes liver organoids function. J. Cell. Biochem. 119 (6), 4320–4333. 10.1002/jcb.26622 29247536

[B66] Saravanan S. Vimalraj S. Thanikaivelan P. Banudevi S. Manivasagam G. (2019). A review on injectable chitosan/beta glycerophosphate hydrogels for bone tissue regeneration. Int. J. Biol. Macromol. 121, 38–54. 10.1016/j.ijbiomac.2018.10.014 30291931

[B67] Seibert C. S. Shinohara E. M. G. Sano-Martins I. S. (2003). *In vitro* hemolytic activity of Lonomia obliqua caterpillar bristle extract on human and Wistar rat erythrocytes. Toxicon 41 (7), 831–839. 10.1016/s0041-0101(03)00040-0 12782083

[B68] She Z. Jin C. Huang Z. Zhang B. Feng Q. Xu Y. (2008). Silk fibroin/chitosan scaffold: Preparation, characterization, and culture with HepG2 cell. J. Mater. Sci. Mater. Med. 19 (12), 3545–3553. 10.1007/s10856-008-3526-y 18622765

[B69] Silva S. S. Motta A. Rodrigues M. T. Pinheiro A. F. Gomes M. E. Mano J. F. (2008). Novel genipin-cross-linked chitosan/silk fibroin sponges for cartilage engineering strategies. Biomacromolecules 9 (10), 2764–2774. 10.1021/bm800874q 18816100

[B70] Song Y. Nagai N. Saijo S. Kaji H. Nishizawa M. Abe T. (2018). *In situ* formation of injectable chitosan-gelatin hydrogels through double crosslinking for sustained intraocular drug delivery. Mater. Sci. Eng. C 88, 1–12. 10.1016/j.msec.2018.02.022 29636124

[B71] Sun W. Incitti T. Migliaresi C. Quattrone A. Casarosa S. Motta A. (2016). Genipin‐crosslinked gelatin–silk fibroin hydrogels for modulating the behaviour of pluripotent cells. J. Tissue Eng. Regen. Med. 10 (10), 876–887. 10.1002/term.1868 24668649

[B72] Supper S. Anton N. Seidel N. Riemenschnitter M. Curdy C. Vandamme T. (2014). Thermosensitive chitosan/glycerophosphate-based hydrogel and its derivatives in pharmaceutical and biomedical applications. Expert Opin. Drug Deliv. 11 (2), 249–267. 10.1517/17425247.2014.867326 24304097

[B73] Tao X. Jiang F. Cheng K. Qi Z. Yadavalli V. K. Lu S. (2021). Synthesis of pH and glucose responsive silk fibroin hydrogels. Int. J. Mol. Sci. 22 (13), 7107. 10.3390/ijms22137107 34281160 PMC8268721

[B74] Thambi T. Phan V. G. Lee D. S. (2016). Stimuli‐sensitive injectable hydrogels based on polysaccharides and their biomedical applications. Macromol. rapid Commun. 37 (23), 1881–1896. 10.1002/marc.201600371 27753168

[B75] Wang L. Neumann M. Fu T. Li W. Cheng X. Su B-L. (2018). Porous and responsive hydrogels for cell therapy. Curr. Opin. Colloid & Interface Sci. 38, 135–157. 10.1016/j.cocis.2018.10.010

[B76] Wang H. Liu H. Liu H. Su W. Chen W. Qin J. (2019). One‐step generation of core–shell gelatin methacrylate (GelMA) microgels using a droplet microfluidic system. Adv. Mater. Technol. 4 (6), 1800632. 10.1002/admt.201800632

[B77] Wu Y. Zhao Z. Guan Y. Zhang Y. (2014). Galactosylated reversible hydrogels as scaffold for HepG2 spheroid generation. Acta biomater. 10 (5), 1965–1974. 10.1016/j.actbio.2013.12.044 24382516

[B78] Wu J. Liu J. Shi Y. Wan Y. (2016). Rheological, mechanical and degradable properties of injectable chitosan/silk fibroin/hydroxyapatite/glycerophosphate hydrogels. J. Mech. Behav. Biomed. Mater. 64, 161–172. 10.1016/j.jmbbm.2016.07.007 27498426

[B79] Yang Q. Zhu Z. Wang L. Xia H. Mao J. Wu J. (2019). The protective effect of silk fibroin on high glucose induced insulin resistance in HepG2 cells. Environ. Toxicol. Pharmacol. 69, 66–71. 10.1016/j.etap.2019.04.001 30959417

[B80] Ye S. Boeter J. W. Penning L. C. Spee B. Schneeberger K. (2019). Hydrogels for liver tissue engineering. Bioengineering 6 (3), 59. 10.3390/bioengineering6030059 31284412 PMC6784004

[B81] Yin Z. Wu F. Xing T. Yadavalli V. K. Kundu S. C. Lu S. (2017). A silk fibroin hydrogel with reversible sol–gel transition. RSC Adv. 7 (39), 24085–24096. 10.1039/c7ra02682j

[B82] Yoshida R. Kaneko Yo Sakai K. Okano T. Sakurai Y. Bae Y. (1994). Positive thermosensitive pulsatile drug release using negative thermosensitive hydrogels. J. Control. Release 32 (1), 97–102. 10.1016/0168-3659(94)90229-1

[B83] Zafar S. Hanif M. Azeem M. Mahmood K. Gondal S. A. (2021). Role of crosslinkers for synthesizing biocompatible, biodegradable and mechanically strong hydrogels with desired release profile. Polym. Bull. 79, 9199–9219. 10.1007/s00289-021-03956-8

[B84] Zhang Y. Lu J. Li Z. Zhu D. Yu X. Li L. (2021). Enhanced cellular functions of hepatocytes in the hyaluronate-alginate-chitosan microcapsules. Int. J. Artif. Organs 44 (5), 340–349. 10.1177/0391398820959345 32969286

[B85] Zhou H. Y. Zhang Y. P. Zhang W. F. Chen X. G. (2011). Biocompatibility and characteristics of injectable chitosan-based thermosensitive hydrogel for drug delivery. Carbohydr. Polym. 83 (4), 1643–1651. 10.1016/j.carbpol.2010.10.022

